# Picroside II protects the blood-brain barrier by inhibiting the oxidative signaling pathway in cerebral ischemia-reperfusion injury

**DOI:** 10.1371/journal.pone.0174414

**Published:** 2017-04-07

**Authors:** Li Zhai, Min Liu, Tingting Wang, Hongyan Zhang, Shan Li, Yunliang Guo

**Affiliations:** 1 Department of Pharmacy, The Third Clinical Medical College of Qingdao University, Qingdao, Shandong Province, China; 2 Institute of Cerebrovascular Disease, Affiliated Hospital of Qingdao University, Qingdao, Shandong Province, China; Indian Institute of Integrative Medicine CSIR, INDIA

## Abstract

**Background and purpose:**

Thrombolysis is used to improve cerebral circulation; at the same time, neuroprotective drugs such as antioxidants should also be used. The aim of these experiments was to explore the protective mechanism of an antioxidant, picroside II, on the blood-brain barrier (BBB) after cerebral ischemia-reperfusion (CI/R) injury.

**Methods:**

To observe the antagonistic effect of picroside II on CI/R damage, the neurological deficit score and the infarct volume were measured. To detect the protective effect of picroside II on nerve cells and the BBB, the morphology and structure of cortical brain tissue were observed, respectively. To investigate the antioxidant effect and mechanism of picroside II, reactive oxygen species (ROS) content, the activity of Nicotinamide adenine dinucleotide phosphate oxidase (NADPH oxidase), and the protein levels of Nox2 and Rac-1 were detected. To investigate the protective mechanism of picroside II on the BBB, the levels of ROCK, MLCK, MMP-2 and claudin-5 were tested.

**Results:**

A higher neurological score, bigger cortex infarction, more damaged neuron structure and injured BBB, increased content of ROS and activity of NADPH oxidase, higher protein levels of Nox2, Rac-1, ROCK, MLCK and MMP-2 and lower levels of claudin-5 were observed in the model group. In the picroside group, the neurological score, neuronal damage, BBB injury, ROS content and NADPH oxidase activity were reduced (*P*<0.05), and the protein levels of Rac-1, Nox2, ROCK, MLCK and MMP-2 were down-regulated (*P*<0.05), while the expression of claudin-5 was up-regulated (*P*<0.05).

**Conclusions:**

Picroside II could protect the nervous system possibly through reducing the content of ROS by down-regulating the expression of Rac-1 and Nox2 and could protect the BBB through reducing the expression of ROCK, MLCK, and MMP-2, while enhancing the expression of claudin-5.

## Introduction

Stroke is a common cerebrovascular emergency, including ischemic stroke and hemorrhagic stroke. Acute ischemic stroke is the most common type of stroke, accounting for approximately 60% to 80% of all strokes[1].

Ischemic stroke is related to the sudden interruption of arterial blood supply, which leads to ischemia and hypoxia of the brain[2]. In most cases, thrombosis is the main cause of ischemic stroke, and rapid ultra-early thrombolytic therapy is the prerequisite and basis for the treatment of acute ischemic stroke. Reperfusion strategies have proven to be the most effective therapies for stroke treatment. However, the restoration of blood flow can lead to reperfusion injury, which causes the breakdown of the blood-brain barrier (BBB) and leads to cerebral hemorrhage and cerebral edema, as well as neurovascular injury and neuronal death, which increases the mortality and disability rate[3]. During ischemia and reperfusion, excessive production of reactive oxygen species (ROS) and reactive nitrogen species (RNS), excitatory amino acid toxicity and the inflammatory reaction are implicated in the neuronal damage of stroke. Among them, oxidative stress is thought to be the primary event during this process because reperfusion stimulates an overproduction of ROS, such as hydrogen peroxide (H_2_O_2_), which leads to the oxidation of proteins, lipids, and DNA and can induce cell proliferation, growth arrest, apoptosis, and necrosis. Under physiological conditions, there is a small amount of ROS that plays an important role in the transmission of physiological signals in the brain[4,5], where a free radical could be offset by antioxidant enzymes. Thus, the generation and elimination of ROS could maintain a balance[6]. However, in the pathological process of cerebral ischemia/reperfusion (CI/R), the restitution of cerebral blood flow re-offers oxygen and glucose, and, at the same time, a series of oxygen-producing enzymes are activated, which leads to the release of a large amount of ROS (O^2-^, H_2_O_2_ and its derivatives)[7,8]. The antioxidant capacity of brain tissue is very fragile, which makes it difficult to resist the attack of free radicals[9]. Moreover, a large number of ROS mediate tissue damage directly, which results in impaired nerve function[10,11,12,13]. Therefore, ROS are believed to be a key cause of the nerve injury after CI/R. Therefore, in order to improve the neurological function of cerebral apoplexy patients, the Guidelines for Diagnosis and Treatment of Acute Ischemic Stroke have noted that neuroprotective drugs that resist ROS must be used in conjunction with thrombolysis therapy.

Free radical scavengers, as neuroprotective drugs, can protect brain cells and increase the tolerance of brain tissue to ischemia and hypoxia in an acute stage of cerebral infarction. Of the antioxidants and free radical scavenging agents, edaravone has been shown to be the most effective, and its clinical effective rate is only 55%, approximately 21.8% higher than placebo[14], indicating that edaravone still has no significant effect on nearly half of patients with stroke. Edaravone is a xanthine oxidase inhibitor, which has an inhibitory effect on active oxygen species[15]. Principal sources of ROS in the brain include the mitochondrial respiratory chain, xanthine oxidase and cyclooxygenase. Recent studies have demonstrated that NADPH oxidases are also important ROS producers. Some studies[16,17]have even suggested that the main producer of ROS is NADPH oxidase and not mitochondria, epoxy synthase or xanthine oxidase[18]. In recent years, *Moskowitz et al*. indicated that NADPH oxidase is the main source of ROS in I/R[19]. Some researchers[20] have also confirmed that NADPH oxidase is the main source of superoxide in the hippocampus. They found that there was almost no peroxide production in neurons without NADPH oxidase. Therefore, the authors concluded that NADPH oxidase inhibitors are likely to be stronger inhibitors of free radicals than edaravone. Therefore, finding a new drug targeting NADPH oxidase could be a good choice for a neuroprotective agent.

Picroside II is the main active ingredient in iridoid glycosides, which is the principal component of *Picrorrhiza kurroa* Royle. Picroside II has antioxidant, anti-inflammatory, immune regulatory, anti-virus and other pharmacological activities[21,22]. Similar to ascorbic acid, picroside II has an excellent antioxidant effect because it contains the molecular structure of a catechol group, which can scavenge oxygen free radicals directly through the reduction reaction. Previous studies have shown that picroside II has a good antioxidant capacity to protect the BBB and can improve the neurological function of rats; thus, it could reduce the death rate of stroke[23,24,25]. However, the molecular mechanism of the antioxidant effect of picroside II is not very clear and needs to be further studied. In this study, the mechanism of antioxidation and protection of the BBB by picroside II in cerebral ischemic reperfusion will be further studied. Our work may provide a better choice for cerebral protection agents.

## Materials and methods

### Materials

The following reagents were used in the study: picroside II (CAS No. 39012-20-9, C_23_H_28_O_13_, 512.48, Tianjin Kuiqing Med. Tech. Co. Ltd.); apocynin (CAS No. 498-02-2, C_9_H_10_O_3_, 166.17, Sigma Aldrich); trans-4-bromine cinnamon acid (TBCA, CAS No. 1200-07-3, C_9_H_7_BrO_2_, 227.05, Sigma Aldrich); 2,3,5-triphenyl tetrazolium chloride (TTC, Chinese Chemical Reagent Co., Ltd.); phenylmethylsulfonyl fluoride (PMSF, no. 329-98-6, Beijing Solarbio Tech. Co. Ltd.); an enhanced BCA protein assay kit (No. P0010, Beyotime Institute of Biotech., China); mouse anti-Rac1 monoclonal antibody (Abcam, ab33186), rabbit anti-Nox2/gp91phox monoclonal antibody (Abcam, ab129068), rabbit anti-ROCK1 monoclonal antibody (Abcam, ab45171), anti-MMP2 (Abcam, ab92536), anti-MLCK (Abcam, ab76092), anti-claudin-5 (Santa Cruz Biotech., Lot# L2013); goat anti-rabbit horseradish peroxidase (HRP)-conjugated secondary antibody (AB136817, Abcam, USA); rabbit anti-β-actin antibody (BA2305, Wuhan Boster Biological Co., Ltd.); rat NADPH oxidase ELISA kit (RG3022) and rat ROS ELISA kit (RG3054) form Trust Specialty Zeal; and Evans Blue (EB, CAS:314-13-16, Sigma Aldrich).

### Animals

All experiments were approved by the Ethics Committee of the Affiliated Hospital of Qingdao University. All animal procedures were conducted in accordance with the guidelines of the National Institutes of Health on the care and use of animals. In total, 280 healthy adult male *Wistar* rats, weighing 240–260 g, were purchased from the Experimental Animal Center of Qingdao Drug Inspection Institute (SCXK(LU)20140010). The animals were housed for 1 week in room temperature and humidity-controlled animal quarters and allowed free access to food and water with natural illumination.

### Rat model of MCAO

Twenty-five rats were randomly selected as the sham operation group, and the other 251 rats were used to established the middle cerebral artery occlusion (MCAO) models by intraluminal insertion of a monofilament suture from the left external-internal carotid artery[26]. Before the operation, all rats were fasted for 12 h, anesthetized with 10% chloral hydrate (3 ml/kg, i.p.), and placed in a prone position on the operating table. A 1.5-cm skin incision along the sagittal suture on the cranial top was made and was wiped with hydrogen peroxide. The anterior fontanelle was exposed, and a hole was made (5 mm left and 3 mm behind bregma) in the dura mater with a dental drill. A Laser Doppler blood flow detector (PE-5001, Sweden) probe was fixed on the dura mater to record the change in the blood flow in the area of the left middle cerebral artery. Once the flow stabilized, the left common carotid artery (CCA), the left external carotid artery (ECA) and the internal carotid artery (ICA) were carefully exposed and isolated. A nylon monofilament surgical thread (18–22 mm) was inserted into the ICA to block the left middle cerebral artery (MCA) when the blunted distal end met resistance. Reperfusion was achieved when the thread was removed after 2 h of occlusion to restore blood supply to the MCA area. The criteria for the inclusion of experimental animals were as follows: The region cerebral blood flow (rCBF) before inserting a monofilament served as the baseline. The rats in which the rCBF fell down to 30% of the baseline or below after inserting the suture and recovered to 80% of the baseline or above after pulling out the monofilament were included.

### Intervention

#### Sham group (n = 5)

The rats in the sham operation group were given the same amount of normal saline 2 h after the operation.

#### Model group (n = 5)

The rats in the model group were given the same amount of normal saline 2 h after the operation (MCAO 2 h).

#### Treatment group (n = 5)

The 1% picroside II solution was formulated with normal saline and administered to the rats at 20 mg/kg by peritoneal injection 2 h after MCAO.

#### Positive control group (n = 5)

The NADPH oxidase-specific inhibitor, apocynin, was diluted with normal saline to make a 2.5% solution, which was administered to rats at 50 mg/kg by peritoneal injection 2 h after MCAO.

#### Treatment + Positive control group (n = 5)

Two hours after ischemia, picroside II (20 mg/kg) and apocynin (50 mg/kg) were administered to rats by peritoneal injection at the same time.

#### Agonist group (n = 5)

The NADPH oxidase activator TBCA, 50 nmol/L in 5 μl of 50% DMSO in PBS, was administered to rats by intracerebroventricular injection 30 minutes before ischemia.

#### Agonist + Treatment group (n = 5)

Thirty minutes before ischemia, TBCA, 5 μl, was administered to rats by intracerebroventricular injection, and 2 h after ischemia, picroside II (20 mg/kg) was administered by peritoneal injection.

#### Vehicle group (n = 5)

The rats were intracerebroventricularly injected with 5 μl of 50% dimethylsulfoxide (DMSO) in PBS 30 minutes before ischemia.

### Evaluation index

#### Neurobehavioral function test

Neurobehavioral function was measured by the modified neurological severity score (mNSS), which contains motor (muscle status and abnormal movement), sensory (tactile, proprioceptive and visual) and reflex tests for a total score of 18. Higher scores indicate more serious neurological function impairments. Each rat was subjected to the test before and after treatment. Multi-group comparisons were made with repeated measures analyses of variance (ANOVA) and multiple comparisons among the different groups, and pairwise comparisons made across time were made using a multivariate ANOVA.

#### Infarct volume assessment

Twenty-two hours after reperfusion, 5 rats were randomly selected form each group and deeply anaesthetized with 10% chloral hydrate (300 mg/kg). The brains of the rats were sectioned into five coronal sections, 2 mm thick. The first cut was located at the middle point of the front pole of the brain and the cross-connecting line, and the second cut was in the chiasma opticum. The third cut was in the infundibular stalk, and the fourth was is between the infundibular stalk and the posterior lobe. Slices were then incubated in 2% TTC phosphate buffer at 37°C for 10 minutes under dark conditions. Normal brain tissue was stained red, and the infarct tissue was white. The Pro Plus 6.0 image system was used to calculate the volume of the cerebral infarction. The infarct volume percentage was expressed by the sum of the infarct area of each section / the sum of the area of each section.

#### HE staining

Twenty-two hours after treatment, five rats from each group were anaesthetized and perfused with 200 ml normal saline and 200 ml 4% paraformaldehyde. Then, the brain was collected and post-fixed in 4% formaldehyde solution for 2 h, dehydrated, clarified, embedded in paraffin, and cut into sections (5 μm in thickness) to adhere to the slide (Leica 2035, Germany). The sections were routinely deparaffinized and stained with hematoxylin eosin (HE). The histological examination was performed under a light microscope; the nuclei in the sections appeared blue, while the cytoplasm appeared red. Five non-overlapping views of the cortex in each section were randomly observed under a microscope (Leica DMI400, Wetzlar, Germany) at 400-fold magnification for cell counting, and the results are presented as the means ± SD. The denatured cell index (the number of denatured cells/total cells) indicates the degree of damage.

#### Evans blue

From each group, five rats were randomly selected to determine the extent of the leakage of the BBB by assessing the extravasation of Evans Blue (EB). Two hours after MCAO, the EB solution (2% wt/vol in sterile PBS) was administered (4 ml/kg) through the left femoral vein. All rats were re-perfused for 22 h after MCAO, and then the brain was taken out to be sliced into 1-mm sections. The BBB damage was visualized by the leakage of EB, which appeared as a blue stain in the brain sections. EB was extracted by homogenizing the samples in 1 ml of 50% trichloroacetic acid solution followed by centrifugation at 12,000 g for 20 minutes. The supernatant was diluted with 100% ethanol at a ratio of 1:3. The amount of EB was quantified at 610 nm by a spectrophotometer (Bio-Tek, Winooski). The leakage rate of EB was expressed as the content of EB in the infarct side / the content of EB in the contralateral hemisphere.

#### Determination of ROS content and NADPH oxidase activity

Twenty-two hours after reperfusion, five rats from each group were randomly selected, anesthetized by chloral hydrate, and then perfused with 200 ml normal saline. A craniotomy was performed to expose the brain, and the residual blood was washed from the brain with normal saline. After removing the apophysis mamillaris and prefrontal brain tissue, the cerebral tissue of the ischemic area was cut backward from the chiasma opticum. Then, the tissue was quickly cut into pieces in Eppendorf tubes, and ice-cold PBS was added to the tube at a proportion of 1:9. The tissue was ground in an ice bath and centrifuged at 4000 rpm for 10 minutes at 4°C in a refrigerated centrifuge (Eppendorf 5801, Germany). After removal of the precipitation and saving the supernatant, the ROS content and NADPH oxidase activity were determined using a ROS ELISA kit and NADPH oxidase ELISA kit. Zero was set with distilled water, and the absorbance was determined at 450 nm to calculate the ROS content and NADPH oxidase activity through a standard curve.

#### Western blot analysis

The brain tissue was ground in an ice bath and centrifuged at 12000 rpm for 10 minutes at 4°C in the refrigerated centrifuge. A 10% SDS-PAGE gel was prepared, and the samples was loaded onto the gel. After electrophoresis, the wet transfer method was used. The membranes were incubated with monoclonal antibodies against Rac-1, Nox2, ROCK, MLCK, MMP-2, claudin-5 and β-actin. Immunoblots were developed using horseradish peroxidase-conjugated secondary antibodies. Immunoreactive bands were visualized by the enhanced chemiluminescent ECL system (Biospectrum 810 Imaging system, UVP, USA) and quantified by densitometry using a ChemiDoc XRS (Bio-Rad, Berkeley, California, USA). The density of each band was normalized to β-actin for their respective lanes.

#### Statistical analysis

The SPSS 17.0 software was used for statistical analysis. One-way analysis of variance (one-way ANOVA) was used for multi-group comparisons. The values were considered significant when *P* was less than 0.05. Statistical differences were determined using a one-way ANOVA followed by Newman-Keuls post hoc tests.

## Results

### Picroside II improved neurological behavioral function (Fig 1)

**Fig 1:**
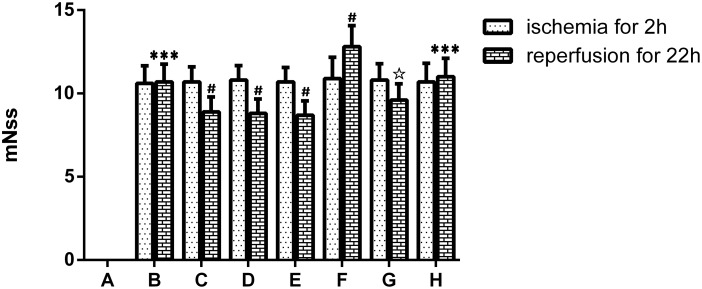
The scores of the neurobehavioral function deficit. A. Sham group; B. Model group; C. Treatment (picroside II) group; D. Positive control (apocynin) group; E. Treatment + Positive control (picroside II + apocynin) group; F. Agonist (TBCA) group; G. Agonist + Treatment (TBCA + picroside II) group; H. Vehicle (DMSO) group. ***P<0.001 compared to group A; #*P*<0.05 compared to group B; ☆*P*<0.05 compared to group F. The data were compared with one-way ANOVA; Values are presented as means ± SD; n = 5.

There were no neurological deficits in the sham group, and the mNSS score was zero. The rats in the model group exhibited neurological deficits, and their mNSS score was significantly higher than that of the sham group (*P*<0.001, Q = 25.069). After treatment with picroside II, the mNSS score was significantly lower than that of the model group (*P*<0.05, Q = 4.217). The scores of the apocynin and picroside II + apocynin groups were also lower than those in the model group (*P*<0.05, Q = 0.234, 0.468), while there was no significant difference among those above three groups before and after treatment, indicating there seems to be no synergistic action of picroside II and apocynin. The score of the TBCA group was obviously higher than that of the model group (*P*<0.05, Q = 4.92), while it was obviously lower after treatment with picroside II (*P*<0.05, Q = 7.497) but still higher than that of the picroside II, apocynin and the cooperative groups (*P*<0.05, Q = 1.64). There was no significant difference between the scores of the vehicle group and the model group (*P*>0.05, Q = 0.702, Fig 1).

### Picroside II decreased the cerebral infarct volume (Fig 2)

**Fig 2:**
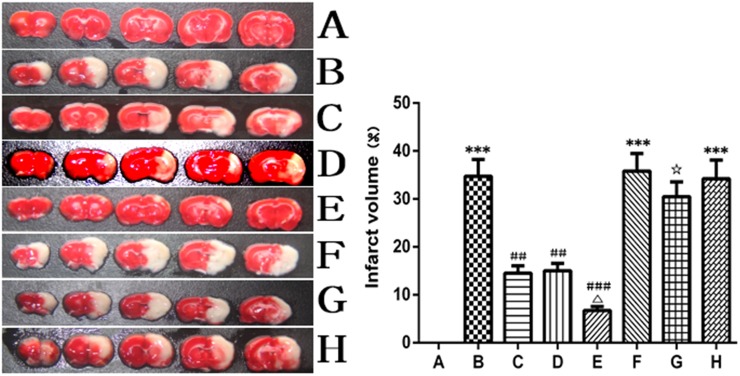
The cerebral ischemic infarct, TTC staining. A. Sham group; B. Model group; C. Treatment (picroside II) group; D. Positive control (apocynin) group; E. Treatment + Positive control (picroside II + apocynin) group; F. Agonist (TBCA) group; G. Agonist + Treatment (TBCA + picroside II) group; H. Vehicle (DMSO) group. ***P<0.001 compared to group A; ## *P*<0.01 compared to group B; ### *P*<0.001 compared to group B; △*P*<0.05 compared to group C; ☆*P*<0.05 compared to group F. The data were compared with one-way ANOVA; Values are presented as means ± SD; n = 5.

There was no cerebral ischemic infarct in the brain sections of sham rats, while large white infarct lesions were observed in the model group (34.75±4.23%), in which the infarct size is related to the cortex, corpus striatum and hippocampus. The infarct volumes of the picroside II (14.12±1.73%), apocynin (13.68±1.38%) and picroside II + apocynin (4.57±0.62%) groups were obviously smaller than those in the model group (*P*<0.05, Q = 17.478, 18.075, 25.152, respectively). Compared with that of the picroside II group, the infarct volume of the picroside II + apocynin group was significantly smaller (*P*<0.05, Q = 0.597), while there was no difference in infarct volume between the picroside II and apocynin groups. After treatment with picroside II, the infarct volume of the TBCA group decreased obviously (*P*<0.05, Q = 4.689), and there was no significant difference between the vehicle group and the model group (*P*>0.05, Q = 0.256, Fig 2).

### Picroside II reduced the deformation of nerve cells (Fig 3)

**Fig 3:**
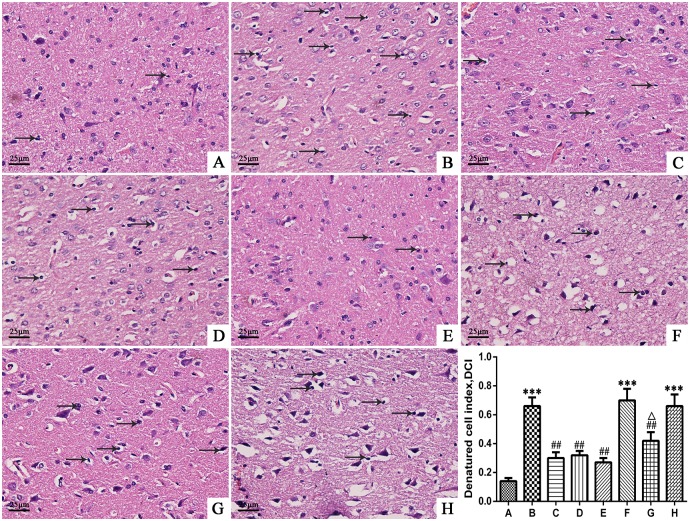
The morphology and structure of neurons in the cortex of rats, HE ×400. A. Sham group; B. Model group; C. Treatment (picroside II) group; D. Positive control (apocynin) group; E. Treatment + Positive control (picroside II + apocynin) group; F. Agonist (TBCA) group; G. Agonist + Treatment (TBCA + picroside II) group; H. Vehicle (DMSO) group. ***P<0.001 compared to group A; ## *P*<0.01 compared to group B; ### *P*<0.001 compared to group B; △△*P*<0.01 compared to group F. The data were compared with one-way ANOVA; Values are presented as means ± SD; n = 5.

HE staining showed that the neurons in the cerebral cortex in the sham group were uniformly distributed and well organized in a complete structure. However, in the model group, TBCA group and DMSO group, the neurons were arranged irregularly with deeply colored and condensed nuclei, karyopyknosis, an increased gap around the neurons, and degenerative changes, such as the formation of vacuoles and necrosis. The denatured cell index (DCI) in the above three groups (0.66±0.06, 0.70±0.08, 0.66±0.08) was much higher than that of the sham group (0.14±0.02, *P*<0.01, Q = 21.318, 22.958, 21.318, respectively). In the picroside II, apocynin, and picroside II + apocynin groups, the neuronal cell damage was only marginal compared to that of the model group; neurons were well arranged with a clear outline, nuclei condensation, and deep coloration observed only in a small number of cells, and there were no significant difference in the DCI values among those three groups (0.30±0.04, 0.32±0.03, 0.27±0.03, respectively, *P*>0.05, Q = 0.820, 2.050, 1.223, respectively), which were much lower than that of the model group (*P*<0.01, Q = 14.759, 13.939, 15.988). In the TBCA + picroside II group, the DCI (0.42±0.06) was significantly lower than that of the TBCA group (*P*<0.01, Q = 11.479), while there was no significant difference between the DCI of the vehicle group and model group (*P*>0.05, Q = 0.00, Fig 3).

### Picroside II inhibited BBB permeability (Fig 4)

**Fig 4:**
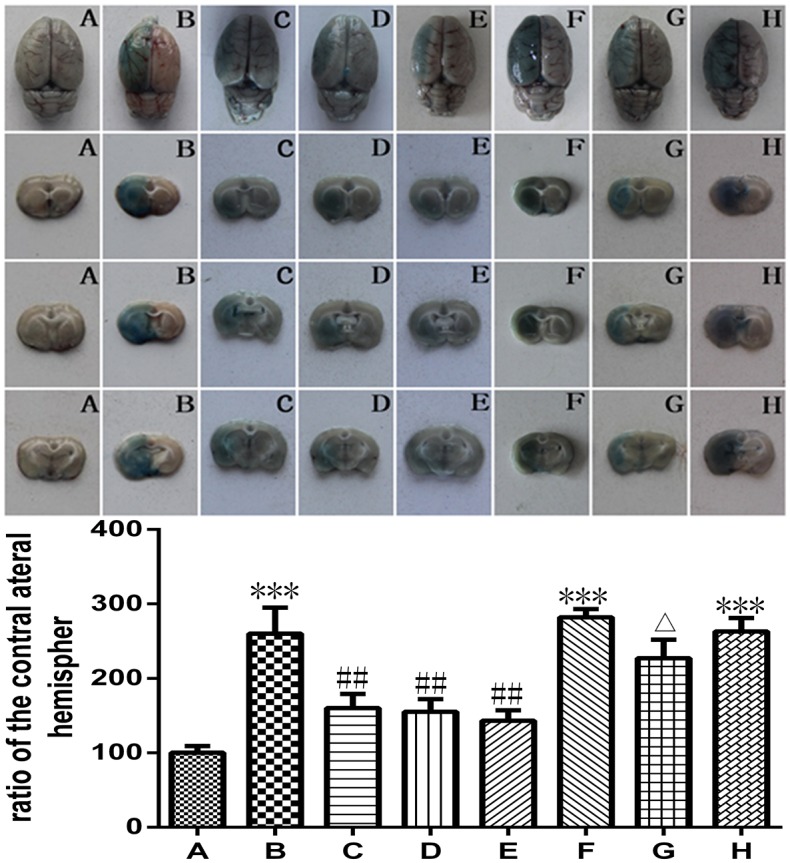
Evans blue leakage rate. A. Sham group; B. Model group; C. Treatment (picroside II) group; D. Positive control (apocynin) group; E. Treatment + Positive control (picroside II + apocynin) group; F. Agonist (TBCA) group; G. Agonist + Treatment (TBCA + picroside II) group; H. Vehicle (DMSO) group. *P<0.05 compared to group B. The data were compared with one-way ANOVA; Values are presented as means ± SD; n = 5.

The extravasation of EB in the left cerebral hemisphere is shown in Fig 4. Compared with that in the sham group, the EB extravasation increased remarkably in response to I/R challenge and was significantly attenuated by treatment with picroside II (*P*<0.05, Q = 9.540). A similar protective effect was observed in the apocynin and picroside II + apocynin group. There seemed to be no significant difference between the treatment effect in the above three groups. The extravasation of EB in the TBCA group was significantly reduced after treatment with picroside II (*P*<0.05, Q = 5.247). The extravasation of EB in the DMSO group was practically at the same level as that in the model group (*P*>0.05, Q = 0.286, Fig 4).

### Picroside II decreased the ROS content and inhibited NADPH oxidase activity (Fig 5)

**Fig 5:**
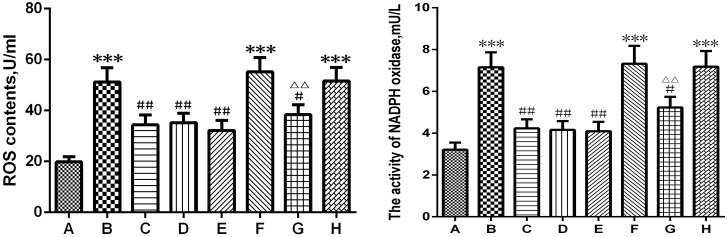
ROS content and NADPH oxidase activity. A. Sham group; B. Model group; C. Treatment (picroside II) group; D. Positive control (apocynin) group; E. Treatment + Positive control (picroside II + apocynin) group; F. Agonist (TBCA) group; G. Agonist + Treatment (TBCA + picroside II) group; H. Vehicle (DMSO) group. *P<0.05 compared to group A; ##*P*<0.01 compared to group B; #*P*<0.05 compared to group B; △△*P*<0.01 compared to group F. The data were compared with one-way ANOVA; Values are presented as means ± SD; n = 5.

Compared with the sham group, the ROS content and NADPH oxidase activity increased significantly in the model group (*P*<0.001, Q = 13.993, 15.264). Picroside II treatment significantly reduced the generation and release of ROS directly by reducing the activity of NADPH oxidase. A similar effect was observed in the apocynin and picroside II + apocynin group (*P*<0.01, Q = 7.50~9.97, Q = 13.19~15.03), and there was no significant difference among those three groups (*P*>0.05, Q = 0.79~2.47, Q = 0.52~1.84). The content of ROS in the TBCA + picroside II group was significantly lower than that in the TBCA group (P<0.05, Q = 9.970). In addition, there was no significant difference between the DMSO and model groups (*P*>0.05, Q = 0.743, Fig 5).

### Picroside II inhibited the expressions of Rac-1 and Nox2 (Fig 6)

**Fig 6:**
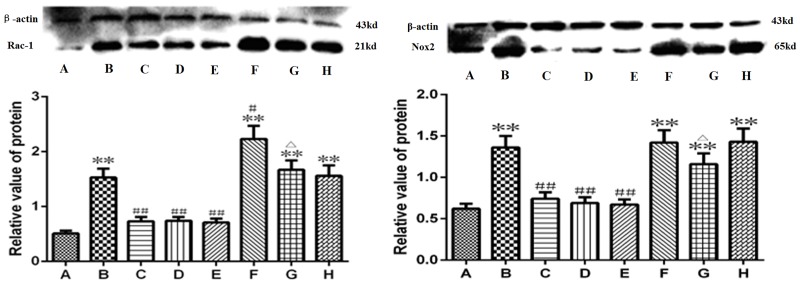
The protein expression level of Rac-1 and Nox2. A. Sham group; B. Model group; C. Treatment group; D. Positive control group; E. Treatment + Positive control group; F. Agonist group; G. Agonist + Treatment group; H. Vehicle group. **P<0.01 compared to group A; *P<0.05 compared to group A; ##*P*<0.01 compared to group B; #*P*<0.05 compared to group B; △*P*<0.05 compared to group F. The data were compared with one-way ANOVA; Values are presented as means ± SD; n = 5.

To test whether picroside II affected the expression of Rac-1 (switch of Nox2) and Nox2 (main producer of ROS), antibodies specific to Rac-1 and Nox2 were used for western blot analysis. The expression levels of Rac-1 and Nox2 were significantly enhanced in the model group. However, in the picroside II, apocynin, and picroside II + apocynin groups, the increased expression of Rac-1 and Nox2 was inhibited (Fig 6). In the TBCA + picroside II group, the expression levels of Rac-1 and Nox2 were significantly lower than those in the TBCA group (*P*<0.05, Q = 4.363, 5.232), while there was no significant difference between the expression levels in the DMSO and model groups (*P*>0.05, Q = 0.301, Fig 6).

### The ROCK/MLCK/MMP-2/claudin-5 pathway was involved in the protective effect of picroside II on the BBB (Fig 7)

**Fig 7:**
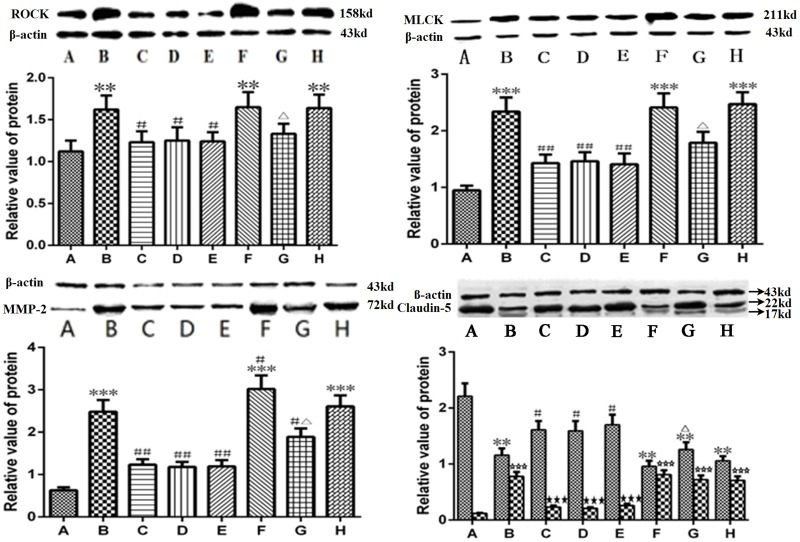
The expression of ROCK, MLCK, MMP-2 and claudin-5. A. Sham group; B. Model group; C. Treatment group; D. Positive control group; E. Treatment + Positive control group; F. Agonist group; G. Agonist + Treatment group; H. Vehicle (DMSO) group. In the lower part of Claudin-5 figure, the first bar is 22KD and the second bar is 17KD in each group. ***P<0.001 compared to group A; **P<0.01 compared to group A; *P<0.05 compared to group A; ##*P*<0.01 compared to group B; #*P*<0.05 compared to group B; △*P*<0.05 compared to group F; ☆☆☆*P*<0.001 compared to group A; ★★★*P*<0.001 compared to group B. The data were compared with one-way ANOVA; Values are presented as means ± SD; n = 5.

ROCK, MLCK, MMP-2 and claudin-5 are important signaling proteins in the damage of the BBB induced by ROS. ROCK and MLCK control endothelial contraction and the reorganization of the cytoskeleton. MMP-2 and claudin-5 are mainly involved in the protein composition of the BBB. We investigated the effects of picroside II on the expression levels of ROCK, MLCK, MMP-2 and claudin-5 in the brains of MCAO rats. The expression levels of ROCK, MLCK, and MMP-2 are shown in Fig 7. The expression levels of ROCK, MLCK and MMP-2 increased in the model group, while they decreased significantly after treatment with picroside II (*P*<0.05, Q = 6.19~7.31), apocynin (*P*<0.01, Q = 6.45~7.83) and picroside + apocynin (*P*<0.01, Q = 6.60~8.15). The expression levels of ROCK, MLCK, and MMP-2 in the TBCA + picroside II group were lower than those in the TBCA group (*P*<0.05, Q = 3.77~6.21).

The molecular weight of claudin-5 is approximately 22 kD. When it degrades, a 17-kD low molecular weight fragment will appear. As shown in Fig 7, the expression of claudin-5 in the model group was significantly lower than in the sham group (*P*<0.01, Q = 14.139), and a 17-kD new band appeared (*P*<0.001, Q = 27.803). However, the expression level of the 22-kD band increased (*P*<0.05, Q = 4.95~8.48) and the 17-kD band decreased (*P*<0.001, Q = 23.73~24.47) in the groups treated with picroside II, apocynin, and picroside II + apocynin. In the TBCA + picroside II group, the expression level of the 22-kD band was higher than that in the TBCA group (*P*<0.01, Q = 4.949), while the expression of the 17-kD band was lower than that in the TBCA group (*P*>0.001, Q = 3.707). There was no significant difference between the expression levels in the DMSO and model group (*P*>0.05, Q = 1.414, 1.854, Fig 7).

## Discussion

In this experiment, we observed that along with an increase in ROS production, the neurological function score and brain infarct volume increased significantly, and the pathology and structure of nerve cells became abnormal after MCAO. After MCAO, rats treated with picroside II, accompanied with a decrease in ROS production, the morphological structure of the nerve cells was ameliorated, and the infarct volume was reduced, while, at the same time, the neurological function recovered significantly. Apocynin and picroside II had a certain synergistic effect in reducing the volume of the cerebral infarction, but there was no obvious synergistic effect on the signaling pathway we have chosen to investigate here. As an activator of Nox2, trans-4-bromine cinnamon acid (TBCA) promoted the generation of ROS. After ventricular injection of TBCA in MCAO rats, the mNSS score increased, and the pathology and structure of nerve cells became abnormal. In the TBCA+ picroside II group, the morphological structure of nerve cells was ameliorated, and the ROS production and the infarct volume were reduced, while, at the same time, the neurological function recovered significantly. These results suggested that picroside II could block or partially block the oxidative activation of TBCA, and then play an antioxidizing role.

ROS are mainly produced by the NADPH oxidase family, which includes Nox1, Nox2, Nox3, Nox4, Nox5, Duox1 and Duox2. Animal experiments[27,28] have proven that Nox2 is the main source of ROS in brain, and its activity and protein level both reflect the ability of brain cells to produce ROS. When the Nox2 gene was deleted, the ability of nerve cells to produce ROS decreased significantly, exhibiting a strong neuroprotective effect[29], such as reducing the infarct volume, alleviating BBB injury and improving neurobehavioral function, etc., but the other members of the NOX family have little effect on ROS production [30]. The activation of Nox2 requires a small GTPase Rac-1, which plays an important role in the aggregation and activation of Nox2 subunits. Rac-1 is the final step in the initiation of Nox2 activity. Our experiments confirmed that after CI/R, the protein levels of Rac-1 and Nox2 in the rat brain increased, and the activity of NADPH oxidase was significantly enhanced. After treatment with picroside II, the protein levels of Rac-1 and Nox2 decreased, and the activity of NADPH oxidase was significantly reduced, which suggested that picroside II exhibited its neuroprotective effect by down-regulating the expression of Rac-1 and Nox2 and by reducing the activity of NADPH oxidase. In the of apocynin and picroside II + apocynin groups, the expression levels of Rac-1 and Nox2 and the activity of NADPH oxidase were not significantly different from that of the picroside II group, which suggested that picroside II and apocynin (an inhibitor of Nox2) exert a similar effect in CI/R, but there is no obvious synergistic effect between picroside II and apocynin. Compared with that of the TBCA group, Rac-1 and Nox2 expression and NADPH oxidase activity were significantly decreased in the TBCA + picroside II group, which suggested that picroside II could block or partially block the effect of TBCA on the activation of the Rac-1/Nox2/ROS pathway.

Another immediate consequence of thrombolytic therapy is the destruction of the BBB, which is the main cause of cerebral hemorrhage and brain edema. The mechanism for this is that cerebral I/R increases ROS[31], and a large number of accumulated ROS change the BBB integrity[32].For example, after co-culturing endothelial cells with ROS, the ROS promote the endothelial cell contraction, which increases the permeability of endothelial monolayers[33]. In the animal model of CI/R, the application of SOD or the antioxidant, tempol, decreased the blood vessel leakage[34,35]. All of the above prove that the role of ROS in the process of BBB damage after CI/R is very important. In these experiments, we have confirmed that, along with the increase in ROS, EB leakage in the brain tissue of rats increased significantly, while the destruction of the BBB resulted in the deformation and shrinkage of neurons. In the picroside II group, with the decrease in the ROS, the EB leakage was significantly reduced, and neuronal injury was significantly alleviated. To explore the mechanism of the protective effect of picroside II on the BBB, we selected ROCK, MLCK, MMP-2, and claudin-5 in the signaling pathway of BBB injury induced by ROS as indicator proteins.

The activation of Nox2 leads to the generation of a large number of ROS, which results in the activation of the Rho-associated kinase pathway (RhoA/ROCK). The activation of ROCK induces the polymerization of actin cytoskeleton, which leads to the destruction of the BBB[36]. Both the generation of ROS and the activation of ROCK result in the activation of myosin light chain kinase (MLCK), which is an important step in BBB dysfunction. MLCK is a substrate-specific kinase that phosphorylates the regulatory light chain (RLC) of myosin II. RLCs then initiate cell contraction and damage the blood brain barrier[37]. Under the combined action of ROCK and MLCK, the cytoskeleton reorganizes to result in the contraction of endothelial cells, which leads to the destruction of the BBB structure. Large amounts of ROS could activate the matrix metalloproteinase (MMP) family. The expression of MMP-2 is significantly increased in the brain blood vessels around the ischemic infarct focus, which could degrade the extracellular matrix, including key vascular proteins such as type IV collagen, laminin, etc., thereby destroying the BBB and resulting in the formation of brain edema[38]. At the same time, MMP-2 can also reduce the expression of claudin-5, which is the constitutive trans-membrane protein of the BBB. All of the above processes harm the structure of the BBB and lead to damage of the BBB[39]. Our experiments confirmed that the expression levels of ROCK, MLCK, and MMP2 were significantly enhanced, while the expression of claudin-5 was significantly decreased after CI/R in rats. After treatment with picroside II, the expression levels of MLCK, ROCK, MMP-2 decreased significantly while claudin-5 (the BBB constitutive protein level) increased obviously. These results suggest that picroside II alleviated BBB damage by decreasing the reorganization of the cytoskeleton and the contraction of the endothelial cells through decreasing the protein levels of ROCK and MLCK, and, at the same time, picroside II developed a protective effect on the BBB by strengthening the structural protein basis of the BBB through reducing the protein expression of MMP-2 and increasing the protein expression of claudin-5.

In conclusion, in the process of CI/R, picroside II exhibited a significant neuroprotective effect via an antioxidant pathway. Picroside II reduced the volume of the cerebral infarct by reducing ROS production through reducing the expression of Rac-1 and Nox2 and the activation of NADPH oxidase, and it alleviated BBB damage by down-regulating the protein levels of ROCK, MLCK, MMP-2 and up-regulating the protein level of claudin-5. These findings suggest that picroside II may have important potential for the development of new agents for the effective treatment of stroke.

## Supporting information

S1 Fig The cerebral ischemic infarct, TTC staining. A. Sham group; B. Model group; C. Treatment (picroside II) group; D. Positive control (apocynin) group; E. Treatment + Positive control (picroside II + apocynin) group; F. Agonist (TBCA) group; G. Agonist + Treatment (TBCA + picroside II) group; H. Vehicle (DMSO) group. ***P<0.001 compared to group A; #*P*<0.05 compared to group B; ☆*P*<0.05 compared to group F. The data were compared with one-way ANOVA; Values are presented as means ± SD; n = 5.
(ZIP)

S2 Fig The morphology and structure of neurons in the cortex of rats, HE ×400. A. Sham group; B. Model group; C. Treatment (picroside II) group; D. Positive control (apocynin) group; E. Treatment + Positive control (picroside II + apocynin) group; F. Agonist (TBCA) group; G. Agonist + Treatment (TBCA + picroside II) group; H. Vehicle (DMSO) group. ***P<0.001 compared to group A; ## *P*<0.01 compared to group B; ### *P*<0.001 compared to group B; △△*P*<0.01 compared to group F. The data were compared with one-way ANOVA; Values are presented as means ± SD; n = 5.
(ZIP)

S3 Fig Evans blue leakage rate. A. Sham group; B. Model group; C. Treatment (picroside II) group; D. Positive control (apocynin) group; E. Treatment + Positive control (picroside II + apocynin) group; F. Agonist (TBCA) group; G. Agonist + Treatment (TBCA + picroside II) group; H. Vehicle (DMSO) group. *P<0.05 compared to group B. The data were compared with one-way ANOVA; Values are presented as means ± SD; n = 5.
(ZIP)

S4 Fig The protein expression level of Rac-1 and Nox2. A. Sham group; B. Model group; C. Treatment group; D. Positive control group; E. Treatment + Positive control group; F. Agonist group; G. Agonist + Treatment group; H. Vehicle group. **P<0.01 compared to group A; *P<0.05 compared to group A; ##*P*<0.01 compared to group B; #*P*<0.05 compared to group B; △*P*<0.05 compared to group F. The data were compared with one-way ANOVA; Values are presented as means ± SD; n = 5.
(RAR)

S5 Fig The expression of ROCK, MLCK, MMP-2 and claudin-5. A. Sham group; B. Model group; C. Treatment group; D. Positive control group; E. Treatment + Positive control group; F. Agonist group; G. Agonist + Treatment group; H. Vehicle (DMSO) group. In the lower part of Claudin-5 figure, the first bar is 22KD and the second bar is 17KD in each group. ***P<0.001 compared to group A; **P<0.01 compared to group A; *P<0.05 compared to group A; ##*P*<0.01 compared to group B; #*P*<0.05 compared to group B; △*P*<0.05 compared to group F; ☆☆☆*P*<0.001 compared to group A; ★★★*P*<0.001 compared to group B. The data were compared with one-way ANOVA; Values are presented as means ± SD; n = 5.
(RAR)
