# Supplementary figures and images for: Picroside II protects the blood-brain barrier by inhibiting the oxidative signaling pathway in cerebral ischemia-reperfusion injury
Source: PLoS One. 2017 Apr 7;12(4):e0174414. doi: 10.1371/journal.pone.0174414 (PMC5384762; doi:10.1371/journal.pone.0174414)

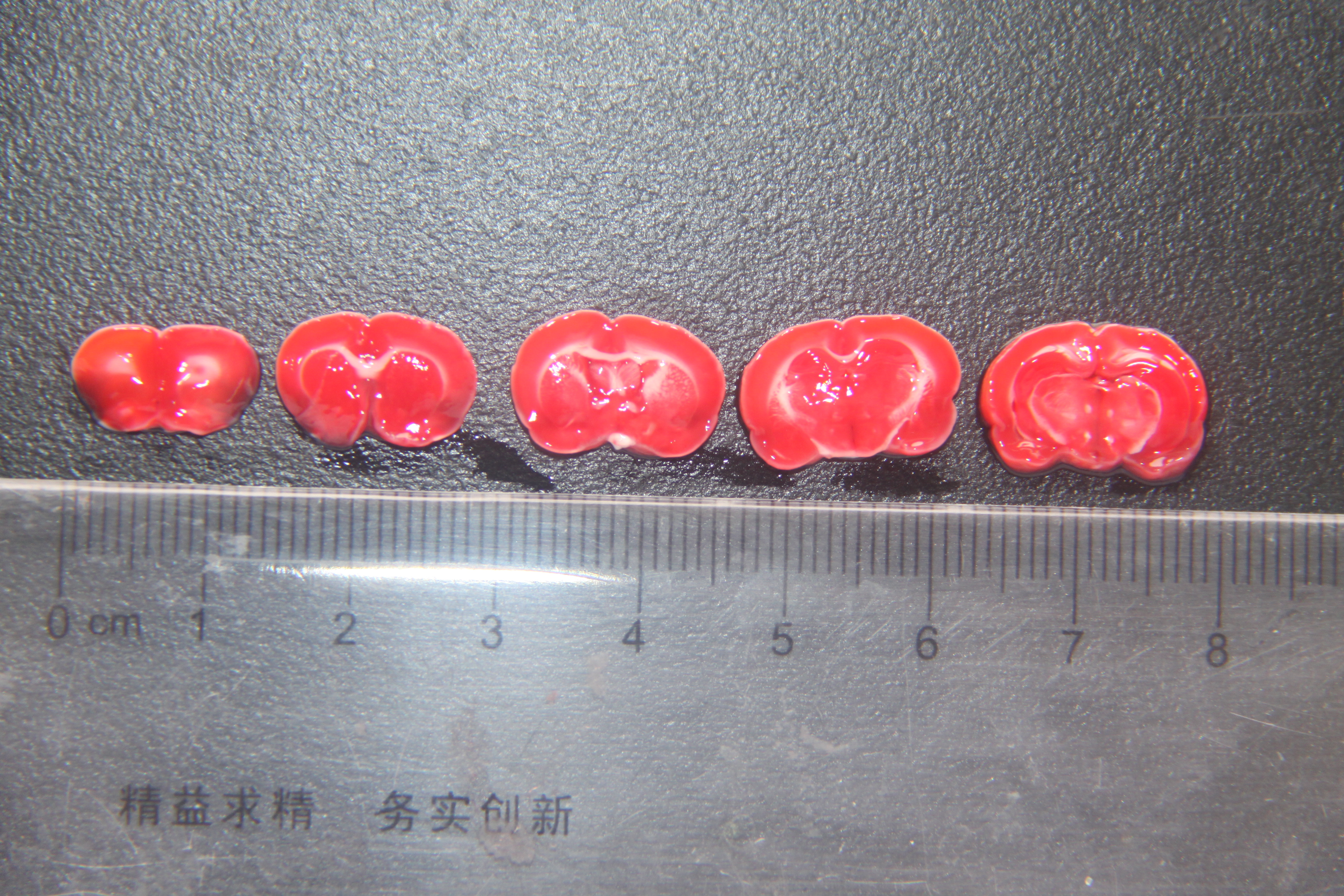

Supplement: S1 Fig — A. Sham group; B. Model group; C. Treatment (picroside II) group; D. Positive control (apocynin) group; E. Treatment + Positive control (picroside II + apocynin) group; F. Agonist (TBCA) group; G. Agonist + Treatment (TBCA + picroside II) group; H. Vehicle (DMSO) group. ***P<0.001 compared to group A; #P<0.05 compared to group B; ☆P<0.05 compared to group F. The data were compared with one-way ANOVA; Values are presented as means ± SD; n = 5. (ZIP) [file pone.0174414.s001.zip › TTC/A.JPG]

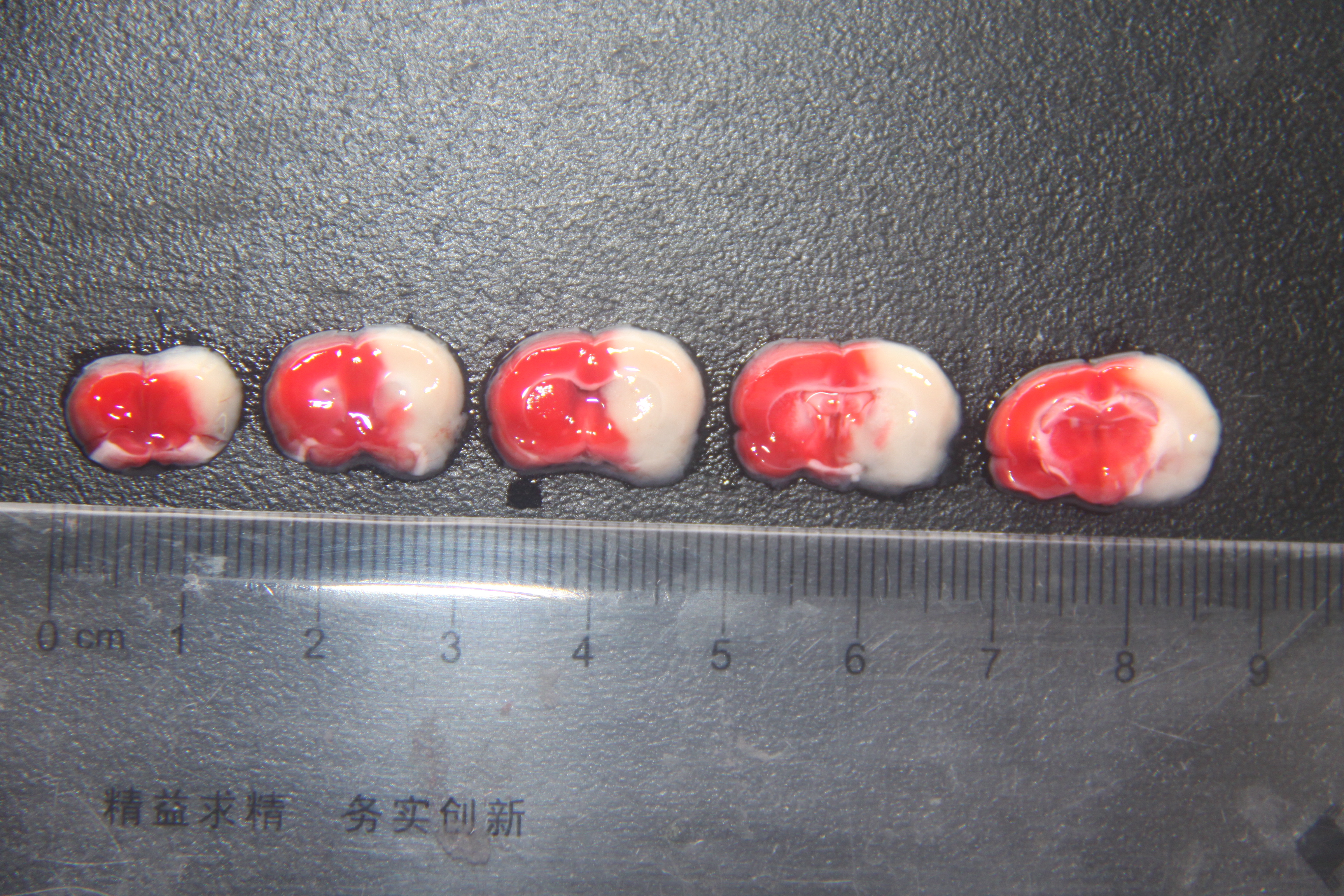

Supplement: S1 Fig — A. Sham group; B. Model group; C. Treatment (picroside II) group; D. Positive control (apocynin) group; E. Treatment + Positive control (picroside II + apocynin) group; F. Agonist (TBCA) group; G. Agonist + Treatment (TBCA + picroside II) group; H. Vehicle (DMSO) group. ***P<0.001 compared to group A; #P<0.05 compared to group B; ☆P<0.05 compared to group F. The data were compared with one-way ANOVA; Values are presented as means ± SD; n = 5. (ZIP) [file pone.0174414.s001.zip › TTC/B.JPG]

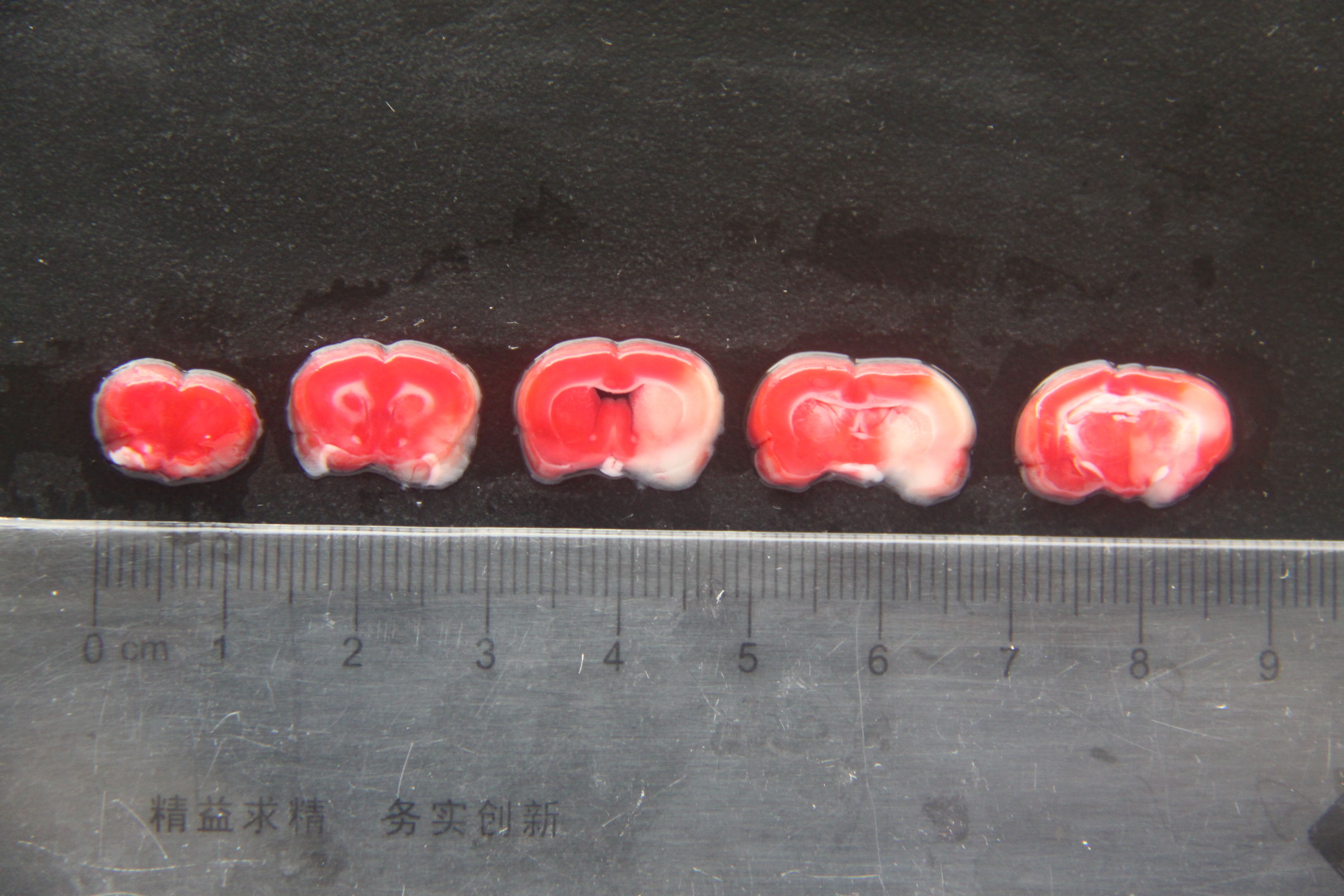

Supplement: S1 Fig — A. Sham group; B. Model group; C. Treatment (picroside II) group; D. Positive control (apocynin) group; E. Treatment + Positive control (picroside II + apocynin) group; F. Agonist (TBCA) group; G. Agonist + Treatment (TBCA + picroside II) group; H. Vehicle (DMSO) group. ***P<0.001 compared to group A; #P<0.05 compared to group B; ☆P<0.05 compared to group F. The data were compared with one-way ANOVA; Values are presented as means ± SD; n = 5. (ZIP) [file pone.0174414.s001.zip › TTC/C.JPG]

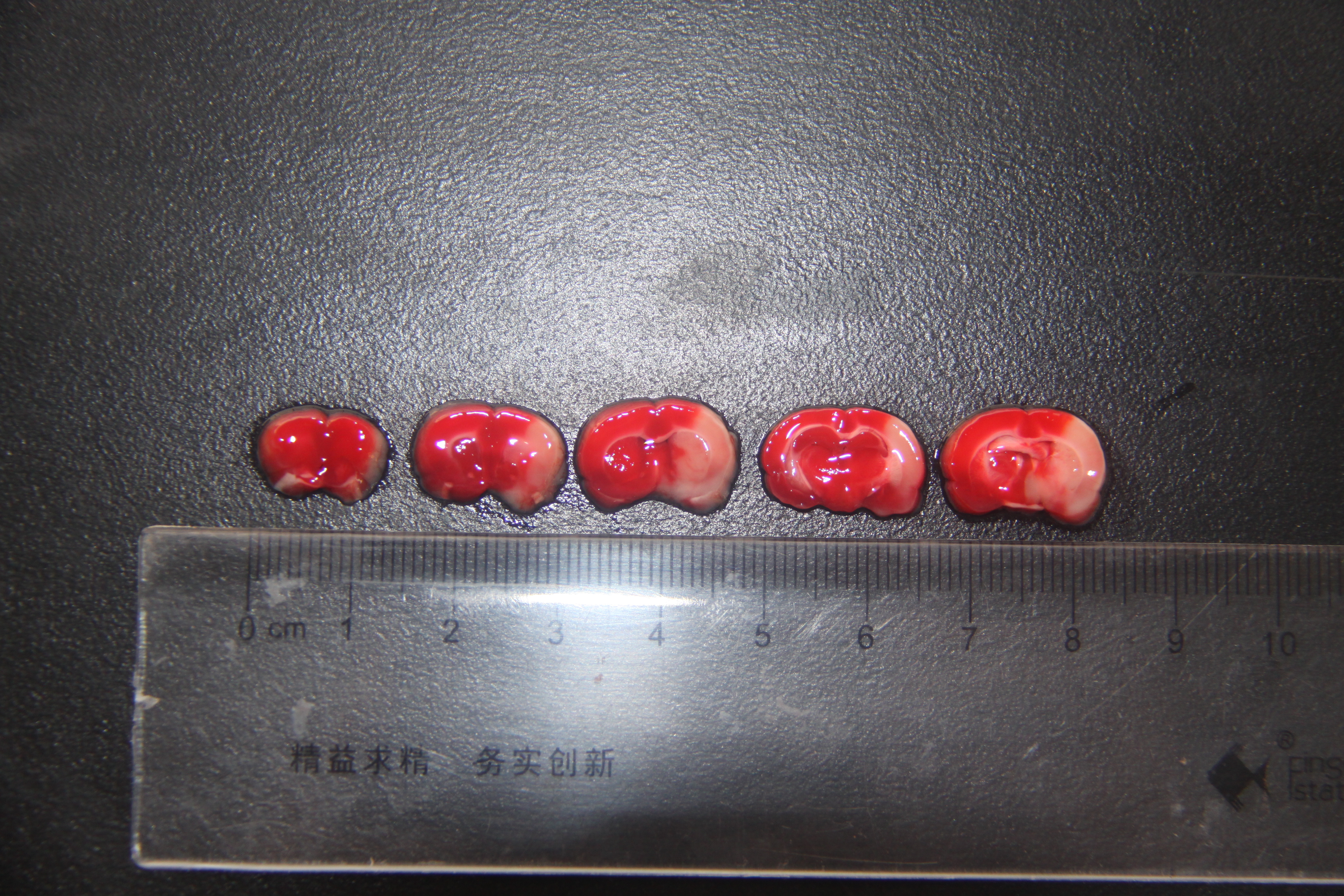

Supplement: S1 Fig — A. Sham group; B. Model group; C. Treatment (picroside II) group; D. Positive control (apocynin) group; E. Treatment + Positive control (picroside II + apocynin) group; F. Agonist (TBCA) group; G. Agonist + Treatment (TBCA + picroside II) group; H. Vehicle (DMSO) group. ***P<0.001 compared to group A; #P<0.05 compared to group B; ☆P<0.05 compared to group F. The data were compared with one-way ANOVA; Values are presented as means ± SD; n = 5. (ZIP) [file pone.0174414.s001.zip › TTC/D.JPG]

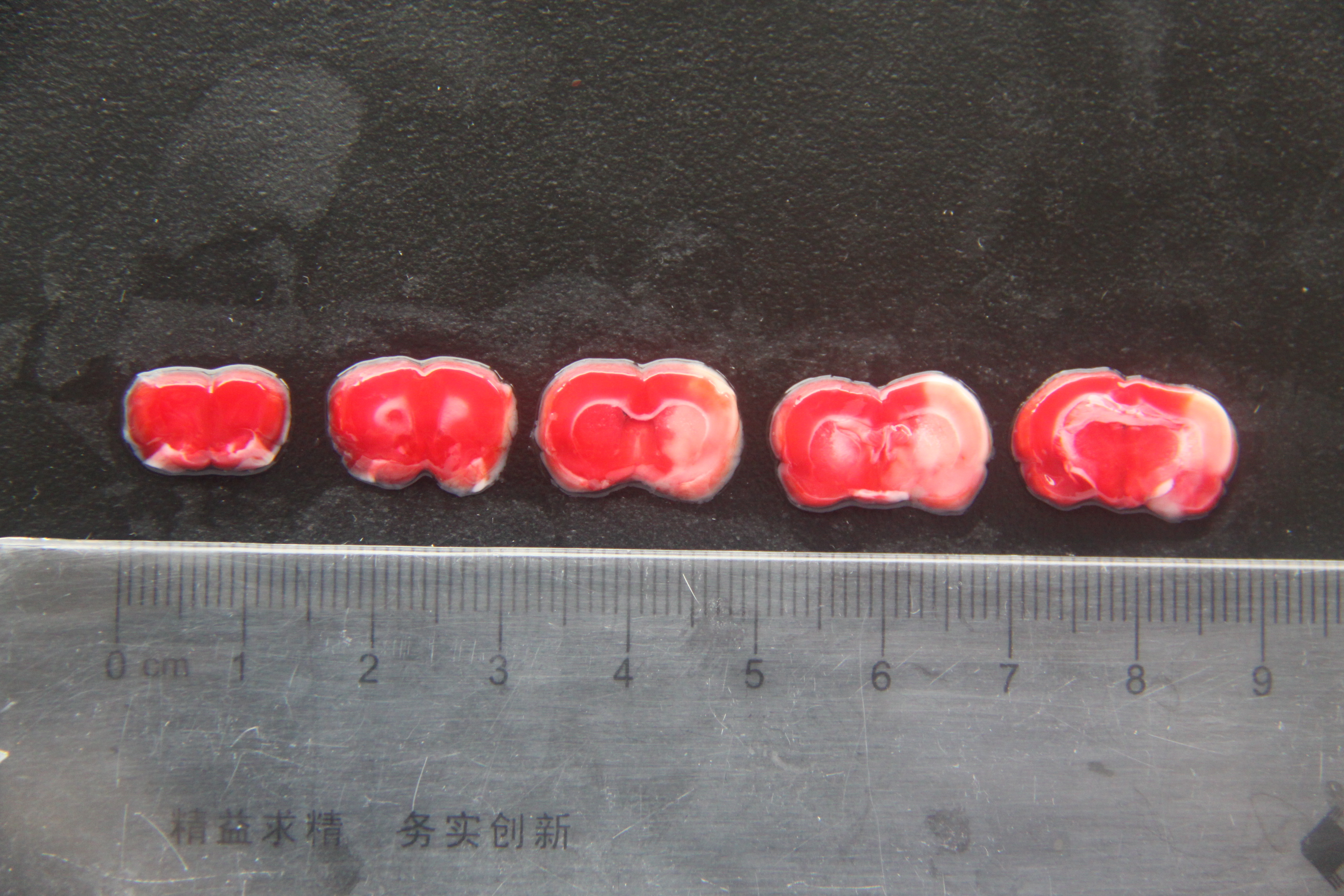

Supplement: S1 Fig — A. Sham group; B. Model group; C. Treatment (picroside II) group; D. Positive control (apocynin) group; E. Treatment + Positive control (picroside II + apocynin) group; F. Agonist (TBCA) group; G. Agonist + Treatment (TBCA + picroside II) group; H. Vehicle (DMSO) group. ***P<0.001 compared to group A; #P<0.05 compared to group B; ☆P<0.05 compared to group F. The data were compared with one-way ANOVA; Values are presented as means ± SD; n = 5. (ZIP) [file pone.0174414.s001.zip › TTC/E.JPG]

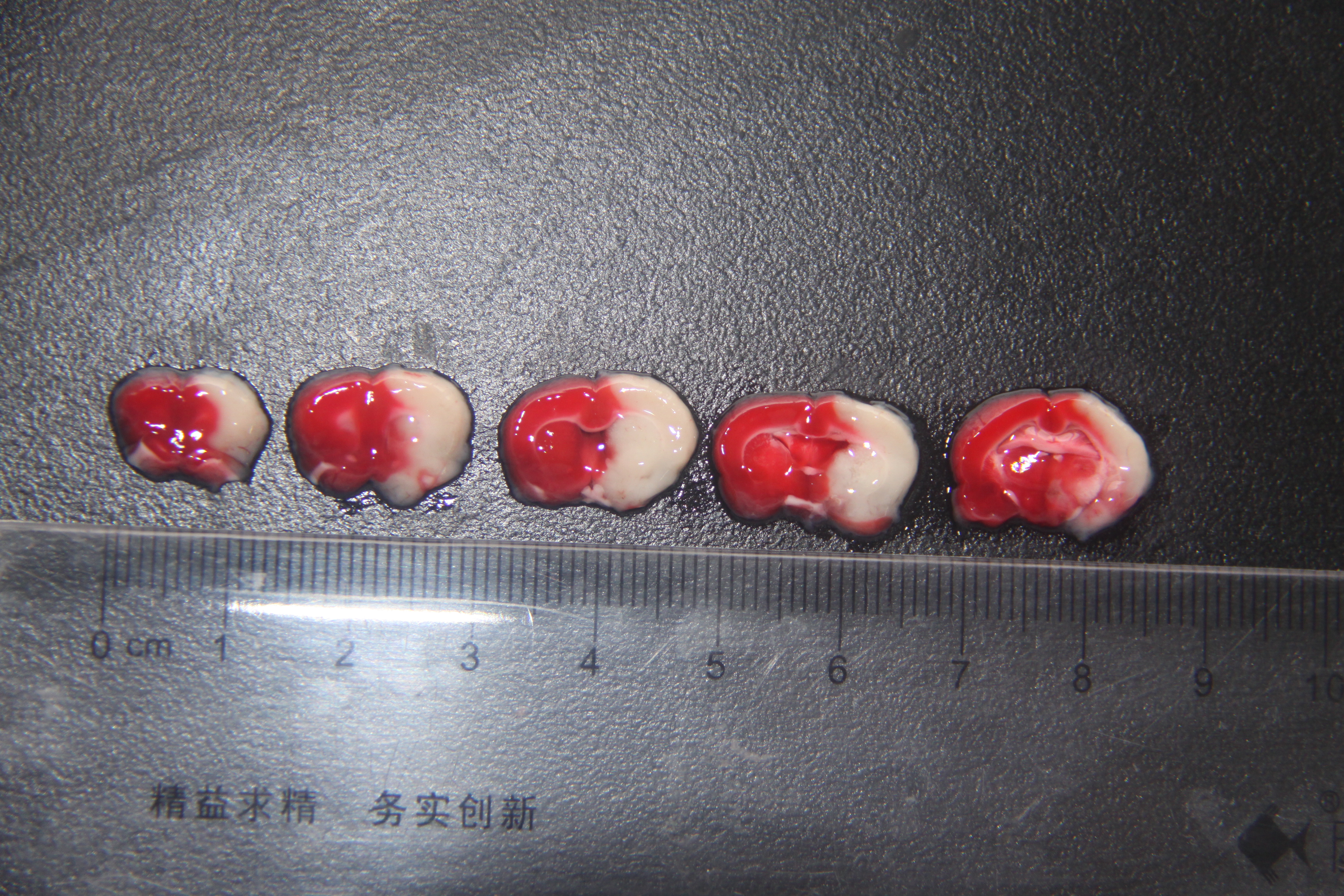

Supplement: S1 Fig — A. Sham group; B. Model group; C. Treatment (picroside II) group; D. Positive control (apocynin) group; E. Treatment + Positive control (picroside II + apocynin) group; F. Agonist (TBCA) group; G. Agonist + Treatment (TBCA + picroside II) group; H. Vehicle (DMSO) group. ***P<0.001 compared to group A; #P<0.05 compared to group B; ☆P<0.05 compared to group F. The data were compared with one-way ANOVA; Values are presented as means ± SD; n = 5. (ZIP) [file pone.0174414.s001.zip › TTC/F.JPG]

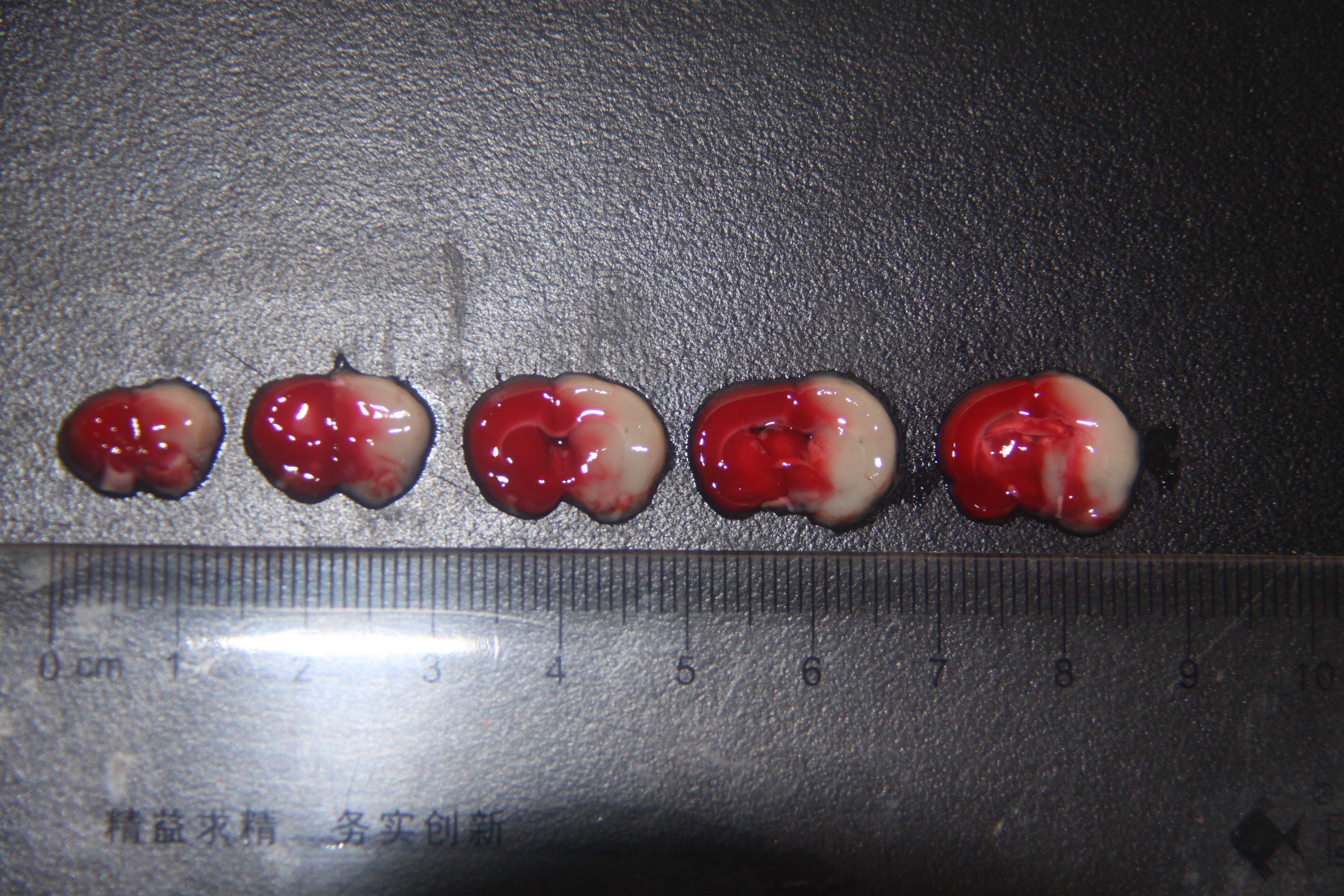

Supplement: S1 Fig — A. Sham group; B. Model group; C. Treatment (picroside II) group; D. Positive control (apocynin) group; E. Treatment + Positive control (picroside II + apocynin) group; F. Agonist (TBCA) group; G. Agonist + Treatment (TBCA + picroside II) group; H. Vehicle (DMSO) group. ***P<0.001 compared to group A; #P<0.05 compared to group B; ☆P<0.05 compared to group F. The data were compared with one-way ANOVA; Values are presented as means ± SD; n = 5. (ZIP) [file pone.0174414.s001.zip › TTC/G.JPG]

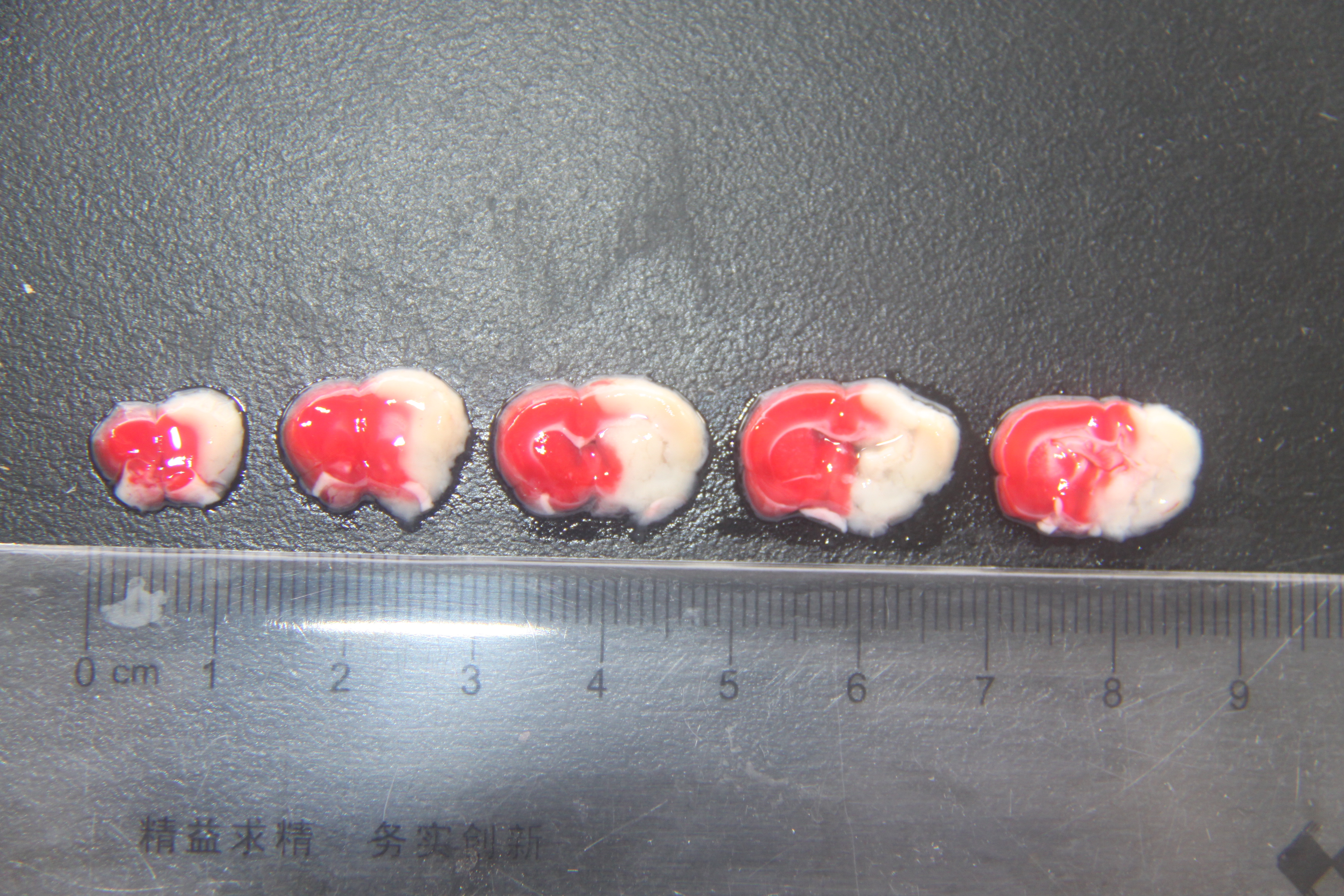

Supplement: S1 Fig — A. Sham group; B. Model group; C. Treatment (picroside II) group; D. Positive control (apocynin) group; E. Treatment + Positive control (picroside II + apocynin) group; F. Agonist (TBCA) group; G. Agonist + Treatment (TBCA + picroside II) group; H. Vehicle (DMSO) group. ***P<0.001 compared to group A; #P<0.05 compared to group B; ☆P<0.05 compared to group F. The data were compared with one-way ANOVA; Values are presented as means ± SD; n = 5. (ZIP) [file pone.0174414.s001.zip › TTC/H.JPG]

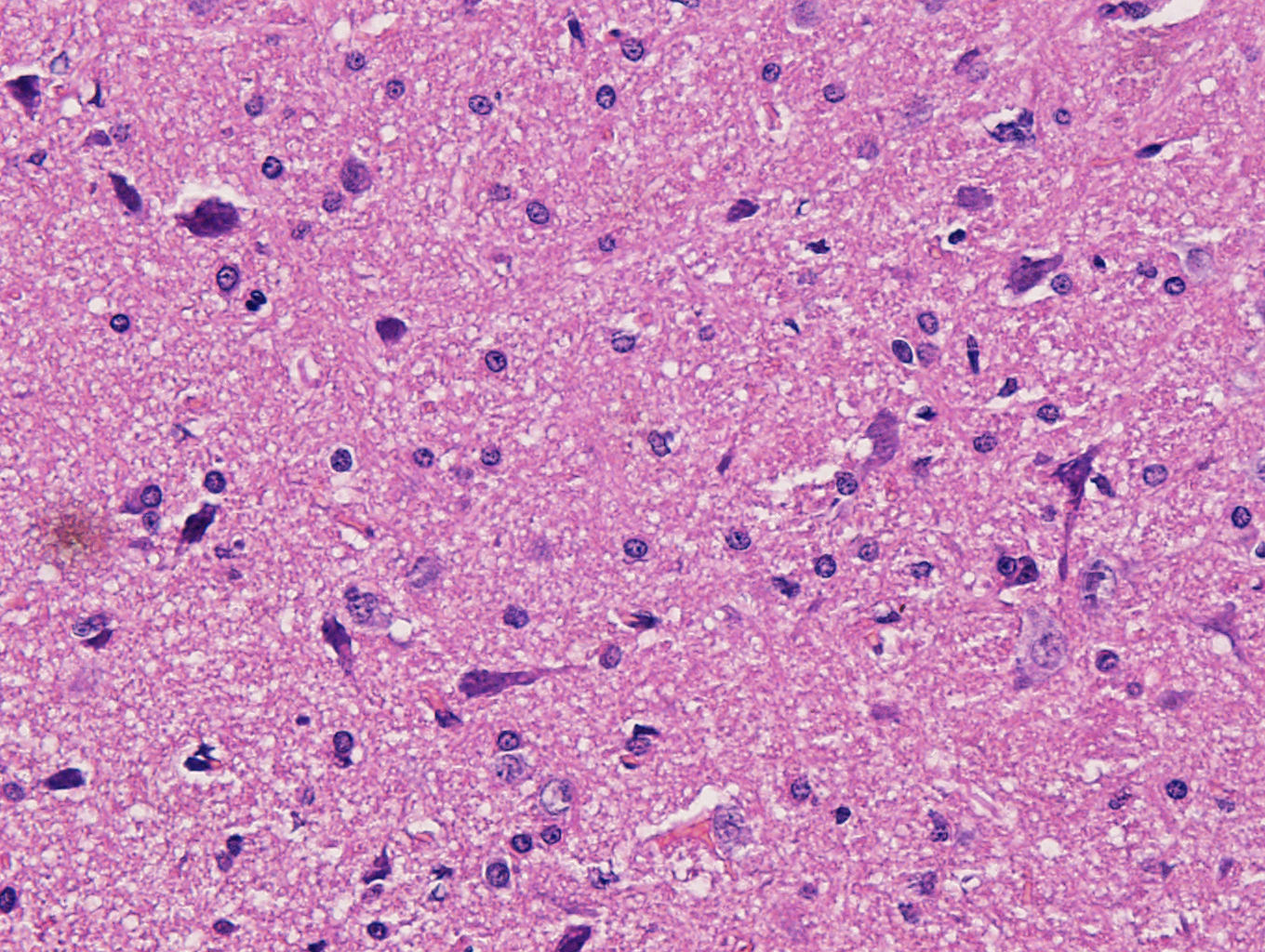

Supplement: S2 Fig — A. Sham group; B. Model group; C. Treatment (picroside II) group; D. Positive control (apocynin) group; E. Treatment + Positive control (picroside II + apocynin) group; F. Agonist (TBCA) group; G. Agonist + Treatment (TBCA + picroside II) group; H. Vehicle (DMSO) group. ***P<0.001 compared to group A; ## P<0.01 compared to group B; ### P<0.001 compared to group B; △△P<0.01 compared to group F. The data were compared with one-way ANOVA; Values are presented as means ± SD; n = 5. (ZIP) [file pone.0174414.s002.zip › HE/A.tif]

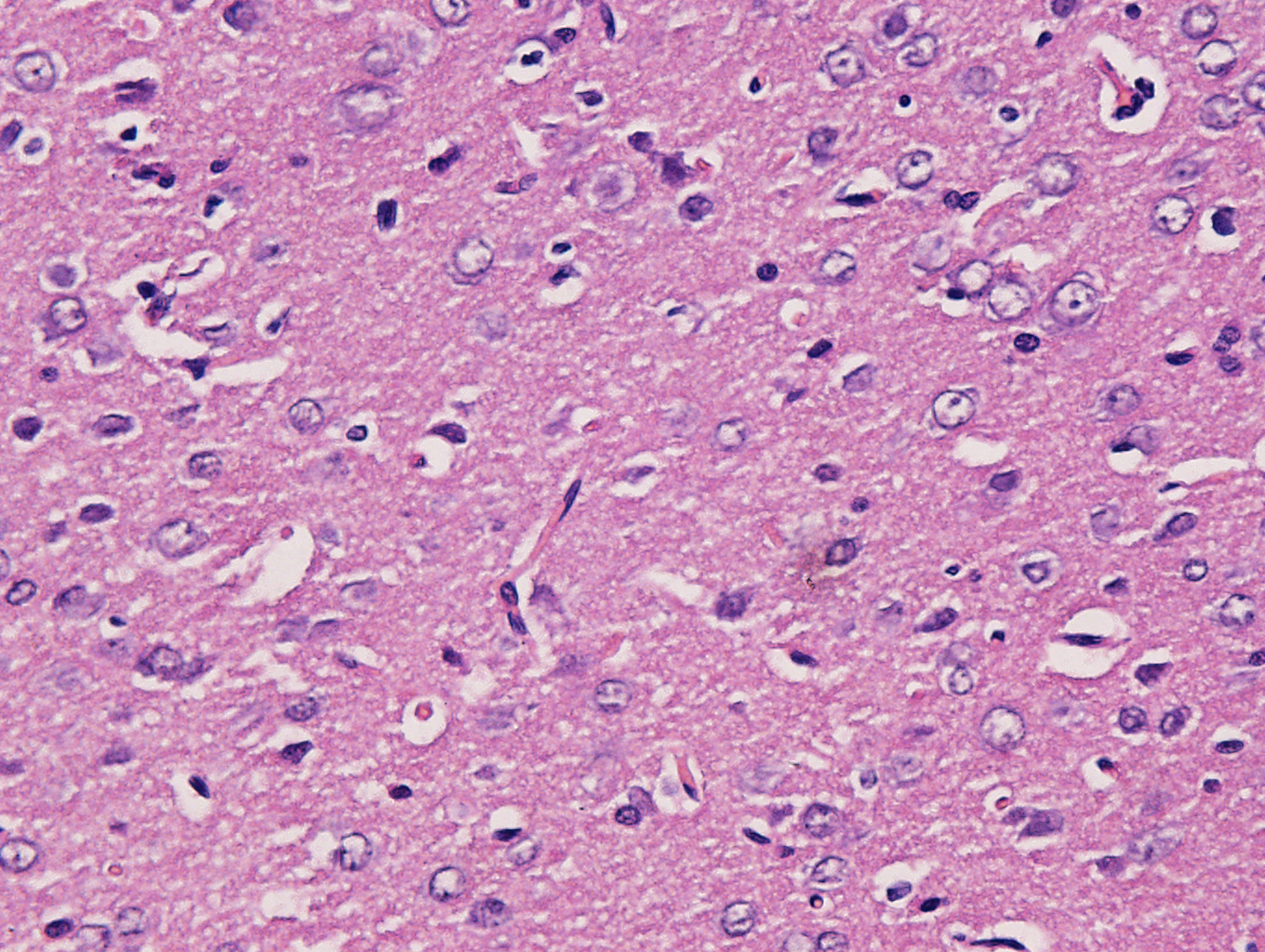

Supplement: S2 Fig — A. Sham group; B. Model group; C. Treatment (picroside II) group; D. Positive control (apocynin) group; E. Treatment + Positive control (picroside II + apocynin) group; F. Agonist (TBCA) group; G. Agonist + Treatment (TBCA + picroside II) group; H. Vehicle (DMSO) group. ***P<0.001 compared to group A; ## P<0.01 compared to group B; ### P<0.001 compared to group B; △△P<0.01 compared to group F. The data were compared with one-way ANOVA; Values are presented as means ± SD; n = 5. (ZIP) [file pone.0174414.s002.zip › HE/B.tif]

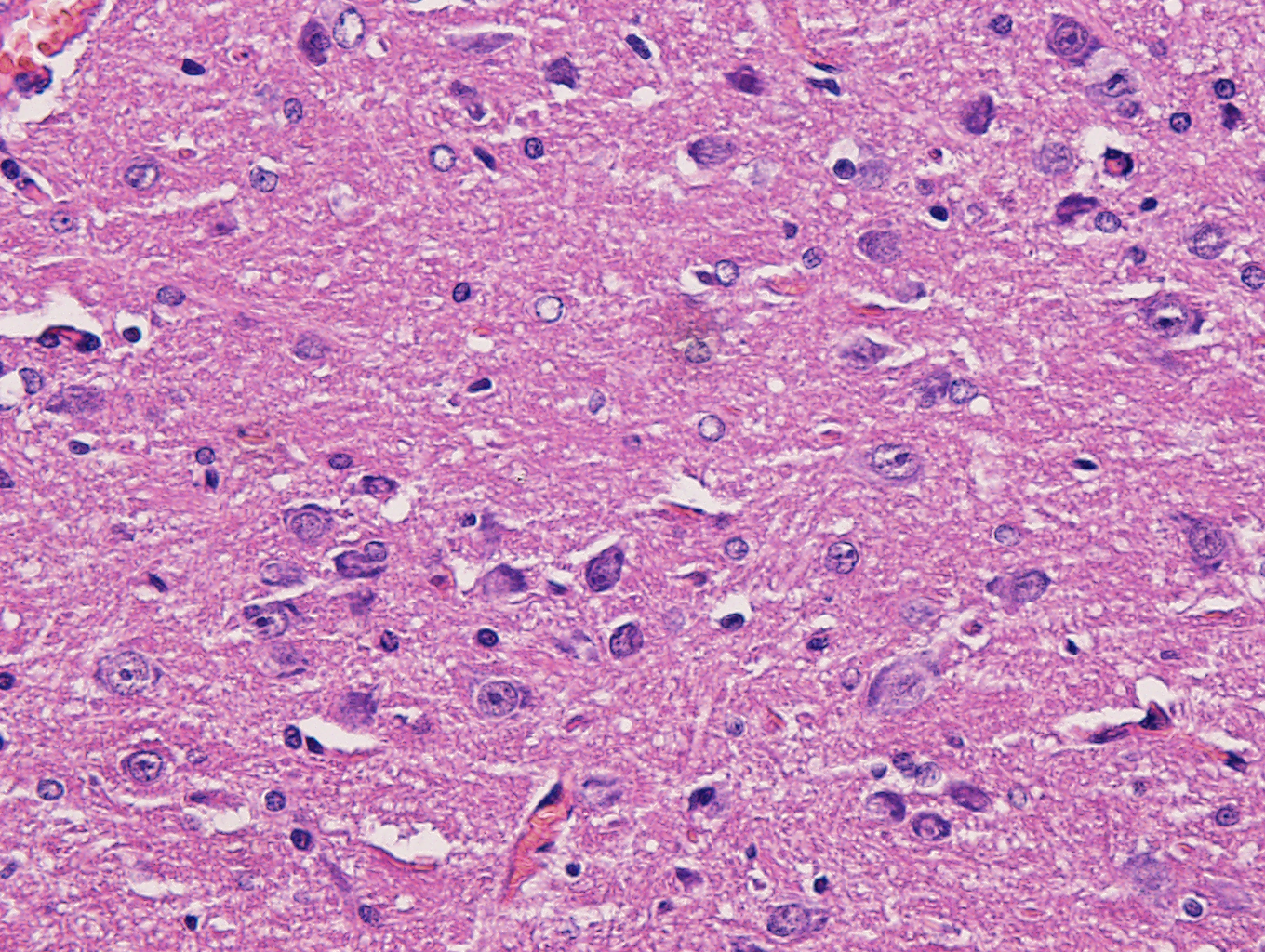

Supplement: S2 Fig — A. Sham group; B. Model group; C. Treatment (picroside II) group; D. Positive control (apocynin) group; E. Treatment + Positive control (picroside II + apocynin) group; F. Agonist (TBCA) group; G. Agonist + Treatment (TBCA + picroside II) group; H. Vehicle (DMSO) group. ***P<0.001 compared to group A; ## P<0.01 compared to group B; ### P<0.001 compared to group B; △△P<0.01 compared to group F. The data were compared with one-way ANOVA; Values are presented as means ± SD; n = 5. (ZIP) [file pone.0174414.s002.zip › HE/C.tif]

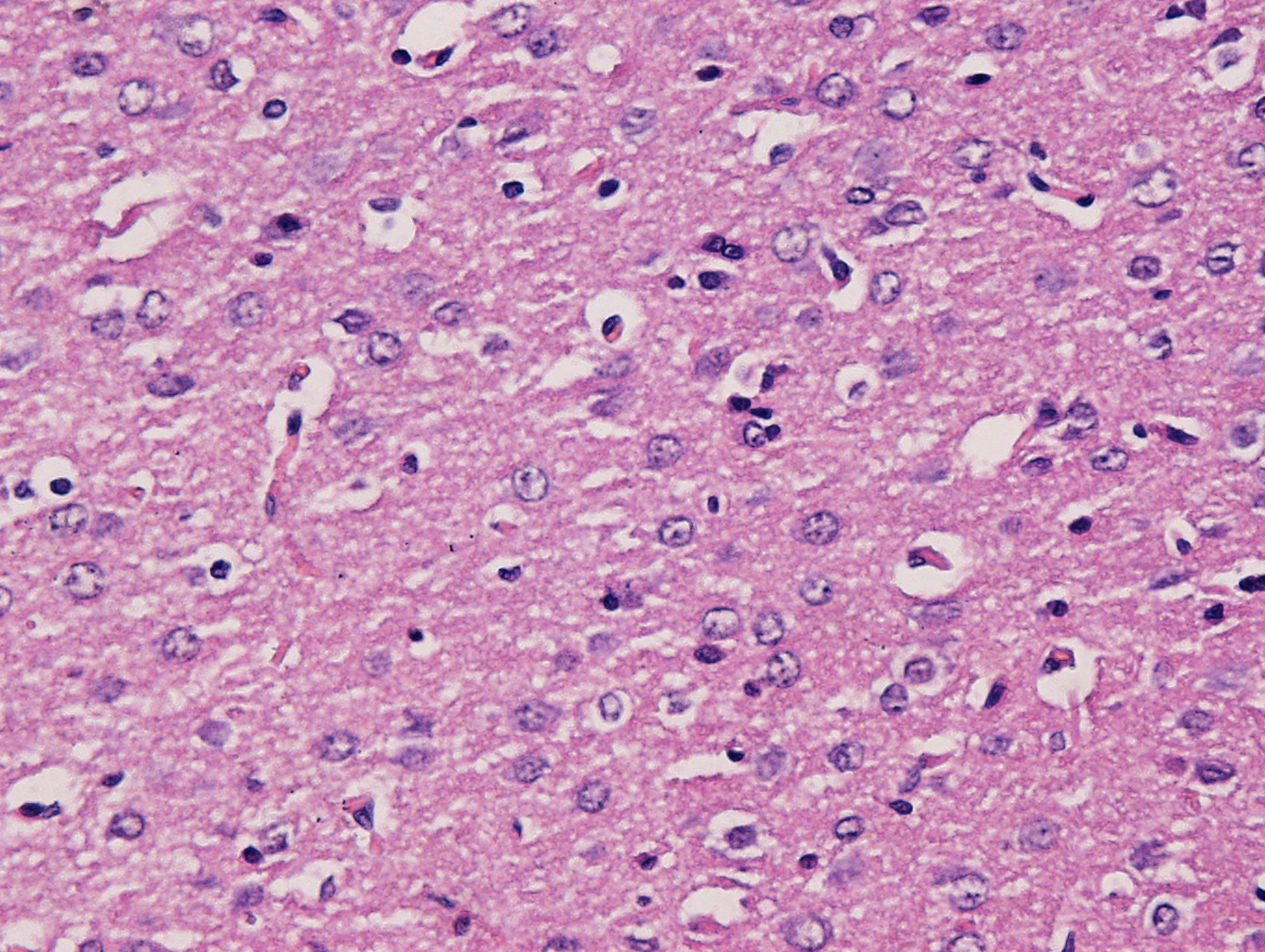

Supplement: S2 Fig — A. Sham group; B. Model group; C. Treatment (picroside II) group; D. Positive control (apocynin) group; E. Treatment + Positive control (picroside II + apocynin) group; F. Agonist (TBCA) group; G. Agonist + Treatment (TBCA + picroside II) group; H. Vehicle (DMSO) group. ***P<0.001 compared to group A; ## P<0.01 compared to group B; ### P<0.001 compared to group B; △△P<0.01 compared to group F. The data were compared with one-way ANOVA; Values are presented as means ± SD; n = 5. (ZIP) [file pone.0174414.s002.zip › HE/D.tif]

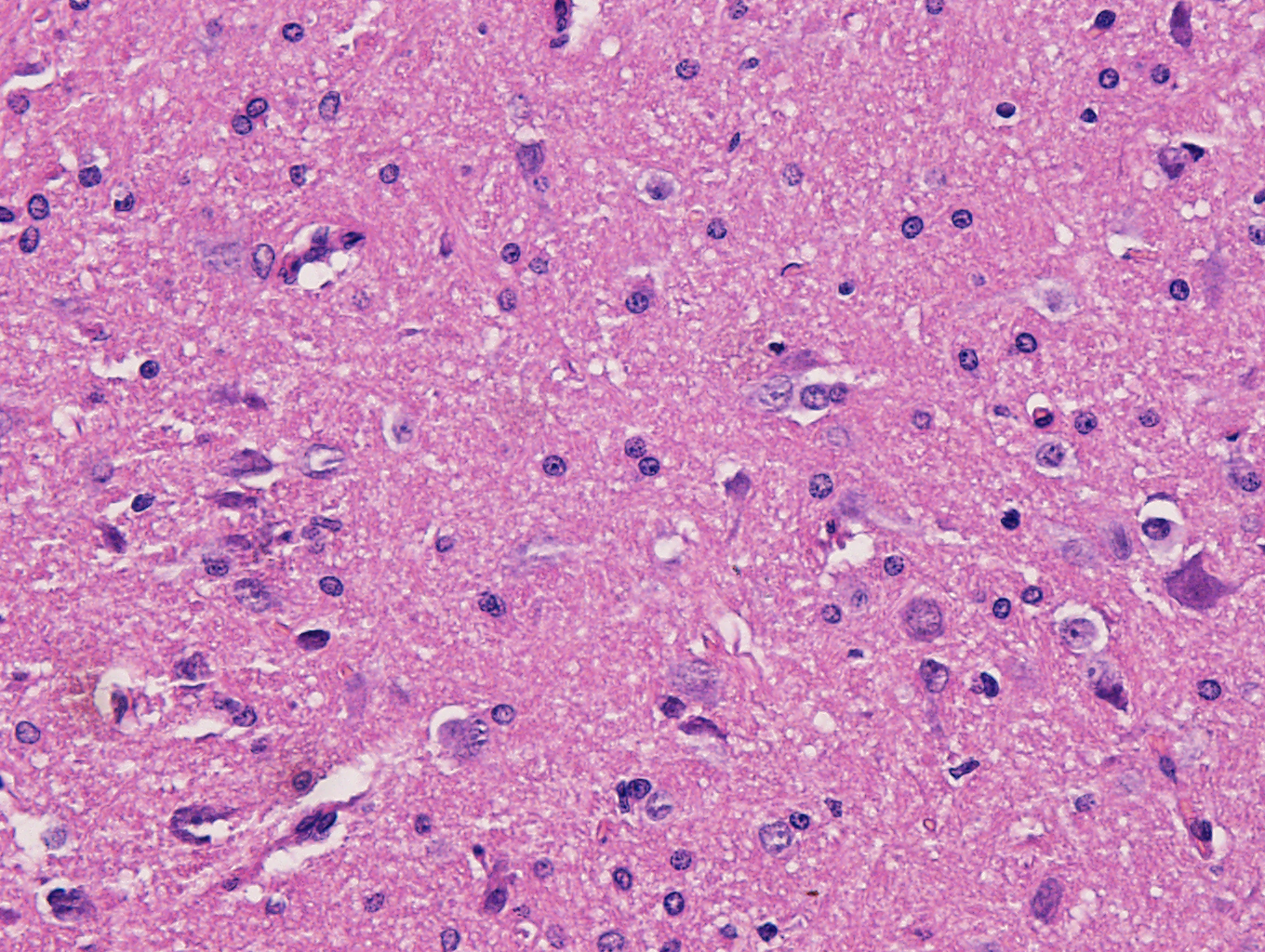

Supplement: S2 Fig — A. Sham group; B. Model group; C. Treatment (picroside II) group; D. Positive control (apocynin) group; E. Treatment + Positive control (picroside II + apocynin) group; F. Agonist (TBCA) group; G. Agonist + Treatment (TBCA + picroside II) group; H. Vehicle (DMSO) group. ***P<0.001 compared to group A; ## P<0.01 compared to group B; ### P<0.001 compared to group B; △△P<0.01 compared to group F. The data were compared with one-way ANOVA; Values are presented as means ± SD; n = 5. (ZIP) [file pone.0174414.s002.zip › HE/E.tif]

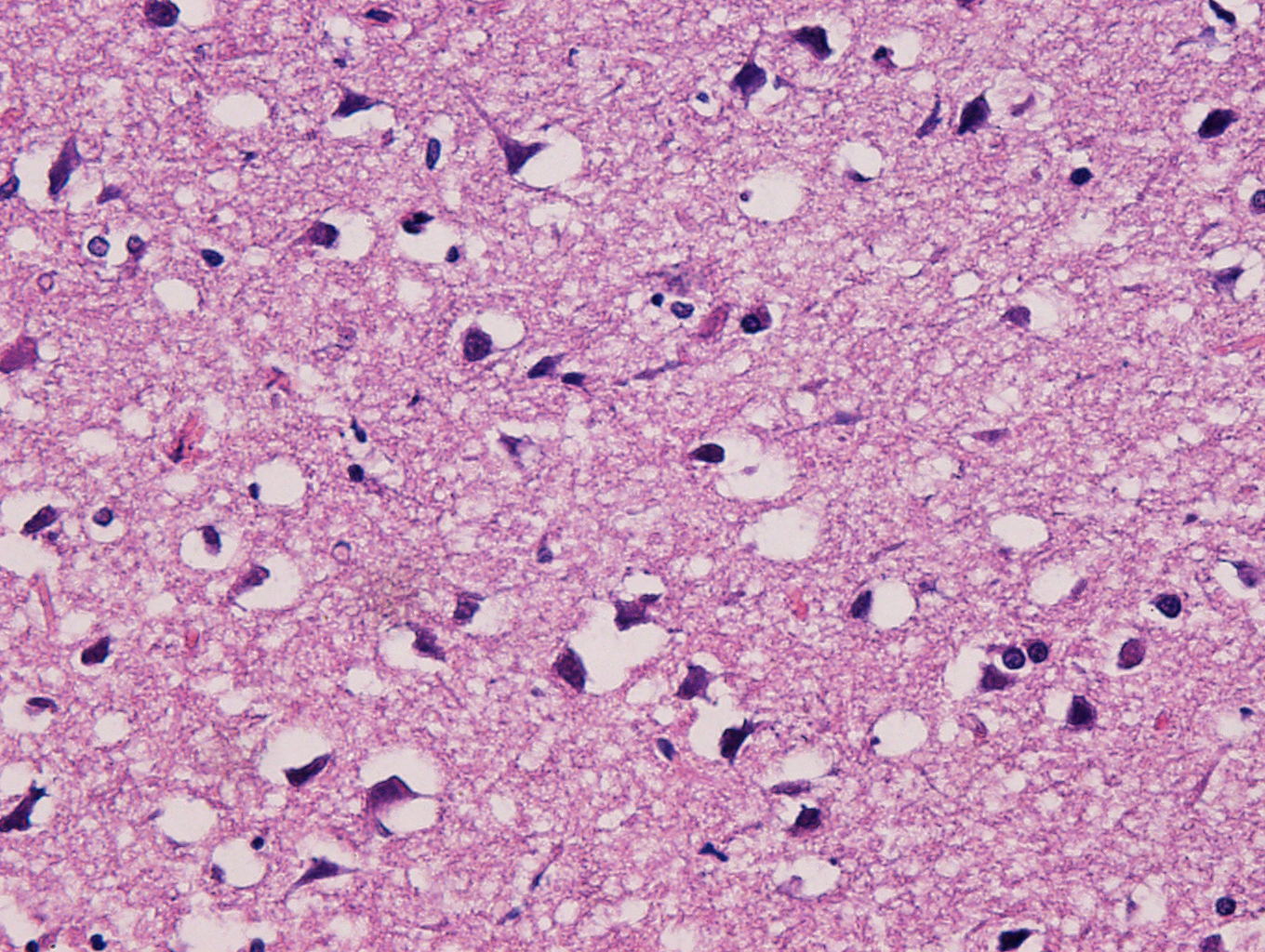

Supplement: S2 Fig — A. Sham group; B. Model group; C. Treatment (picroside II) group; D. Positive control (apocynin) group; E. Treatment + Positive control (picroside II + apocynin) group; F. Agonist (TBCA) group; G. Agonist + Treatment (TBCA + picroside II) group; H. Vehicle (DMSO) group. ***P<0.001 compared to group A; ## P<0.01 compared to group B; ### P<0.001 compared to group B; △△P<0.01 compared to group F. The data were compared with one-way ANOVA; Values are presented as means ± SD; n = 5. (ZIP) [file pone.0174414.s002.zip › HE/F.tif]

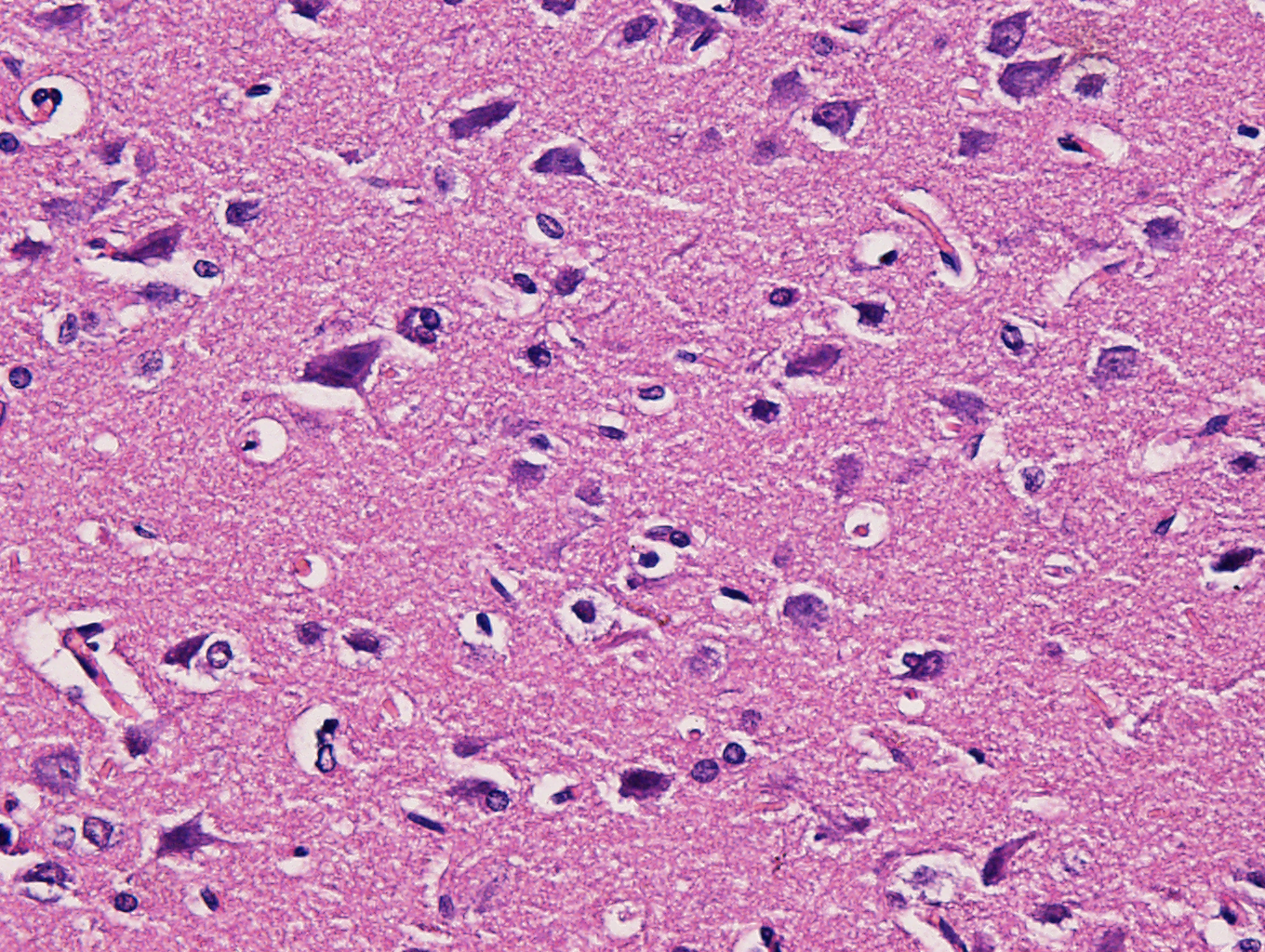

Supplement: S2 Fig — A. Sham group; B. Model group; C. Treatment (picroside II) group; D. Positive control (apocynin) group; E. Treatment + Positive control (picroside II + apocynin) group; F. Agonist (TBCA) group; G. Agonist + Treatment (TBCA + picroside II) group; H. Vehicle (DMSO) group. ***P<0.001 compared to group A; ## P<0.01 compared to group B; ### P<0.001 compared to group B; △△P<0.01 compared to group F. The data were compared with one-way ANOVA; Values are presented as means ± SD; n = 5. (ZIP) [file pone.0174414.s002.zip › HE/G.tif]

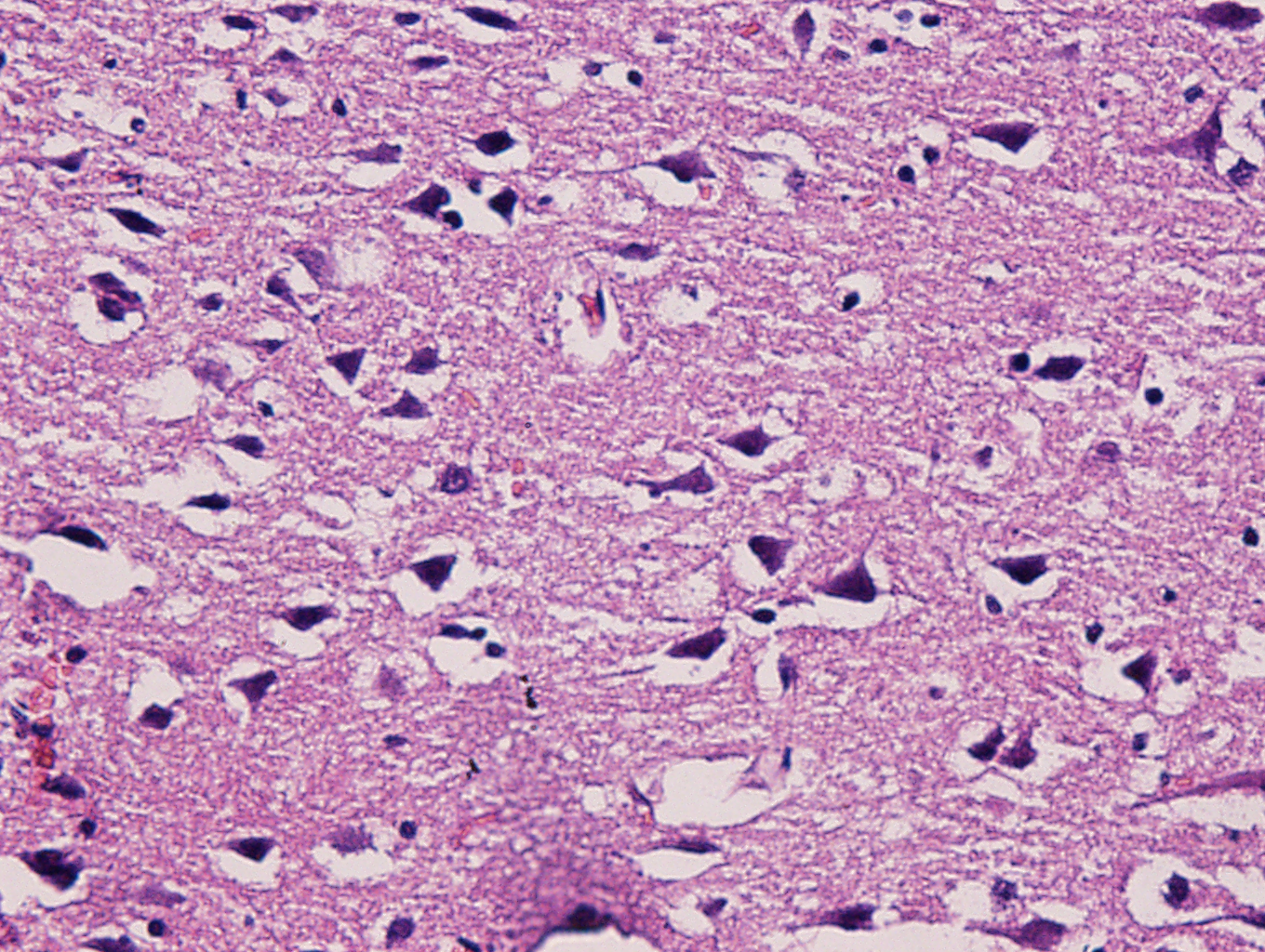

Supplement: S2 Fig — A. Sham group; B. Model group; C. Treatment (picroside II) group; D. Positive control (apocynin) group; E. Treatment + Positive control (picroside II + apocynin) group; F. Agonist (TBCA) group; G. Agonist + Treatment (TBCA + picroside II) group; H. Vehicle (DMSO) group. ***P<0.001 compared to group A; ## P<0.01 compared to group B; ### P<0.001 compared to group B; △△P<0.01 compared to group F. The data were compared with one-way ANOVA; Values are presented as means ± SD; n = 5. (ZIP) [file pone.0174414.s002.zip › HE/H.tif]

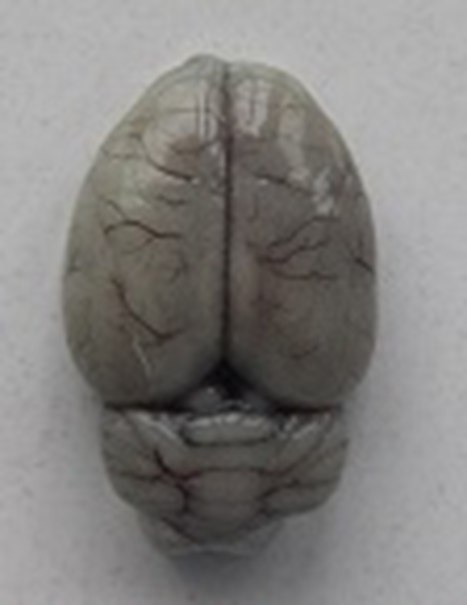

Supplement: S3 Fig — A. Sham group; B. Model group; C. Treatment (picroside II) group; D. Positive control (apocynin) group; E. Treatment + Positive control (picroside II + apocynin) group; F. Agonist (TBCA) group; G. Agonist + Treatment (TBCA + picroside II) group; H. Vehicle (DMSO) group. *P<0.05 compared to group B. The data were compared with one-way ANOVA; Values are presented as means ± SD; n = 5. (ZIP) [file pone.0174414.s003.zip › S3 Fig/A1.tif]

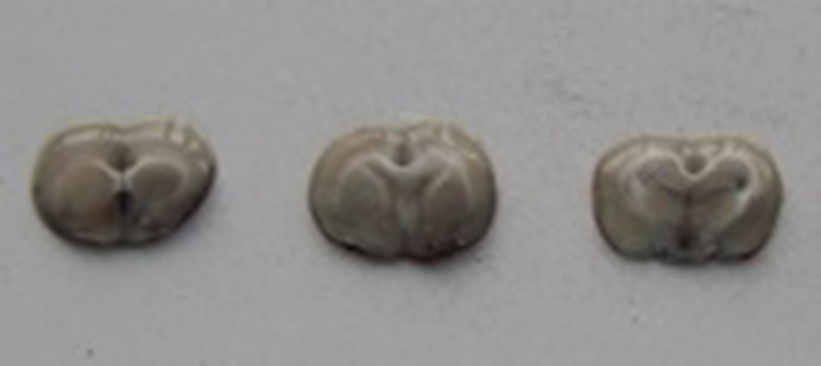

Supplement: S3 Fig — A. Sham group; B. Model group; C. Treatment (picroside II) group; D. Positive control (apocynin) group; E. Treatment + Positive control (picroside II + apocynin) group; F. Agonist (TBCA) group; G. Agonist + Treatment (TBCA + picroside II) group; H. Vehicle (DMSO) group. *P<0.05 compared to group B. The data were compared with one-way ANOVA; Values are presented as means ± SD; n = 5. (ZIP) [file pone.0174414.s003.zip › S3 Fig/A2.tif]

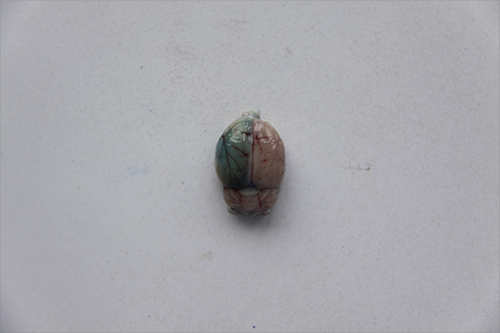

Supplement: S3 Fig — A. Sham group; B. Model group; C. Treatment (picroside II) group; D. Positive control (apocynin) group; E. Treatment + Positive control (picroside II + apocynin) group; F. Agonist (TBCA) group; G. Agonist + Treatment (TBCA + picroside II) group; H. Vehicle (DMSO) group. *P<0.05 compared to group B. The data were compared with one-way ANOVA; Values are presented as means ± SD; n = 5. (ZIP) [file pone.0174414.s003.zip › S3 Fig/B1.tif]

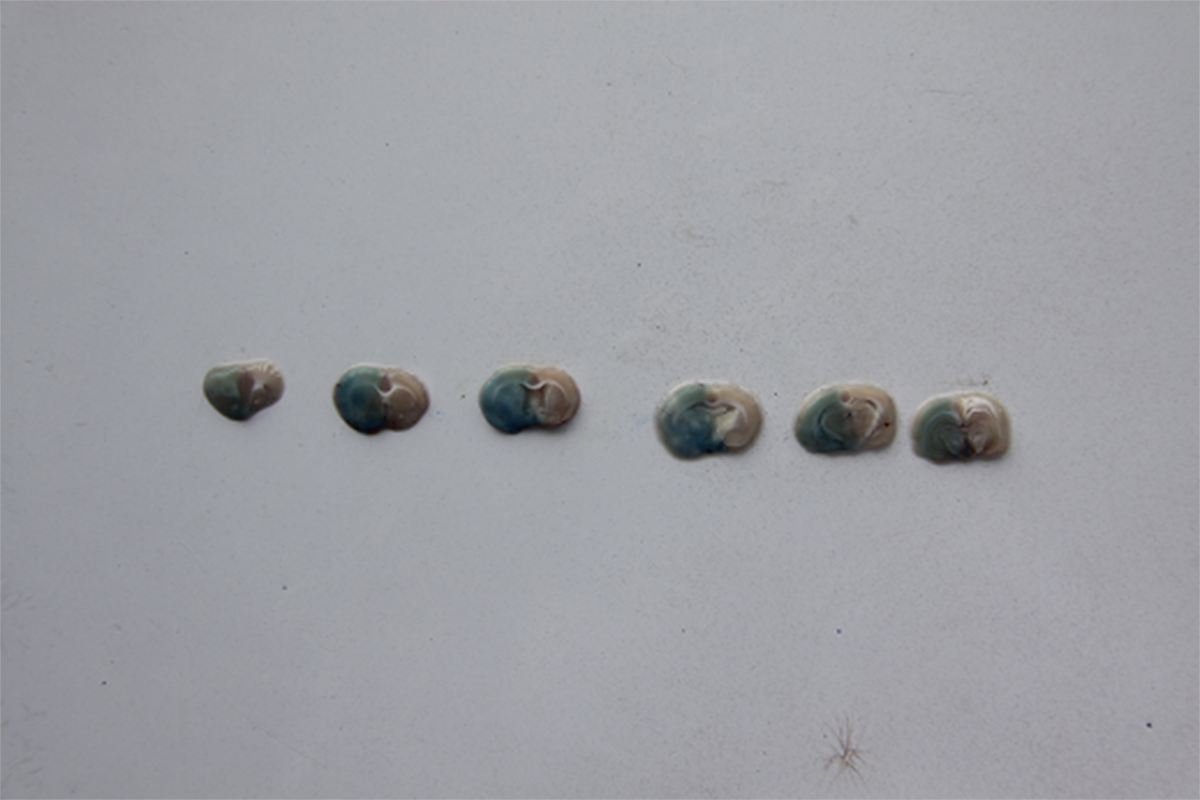

Supplement: S3 Fig — A. Sham group; B. Model group; C. Treatment (picroside II) group; D. Positive control (apocynin) group; E. Treatment + Positive control (picroside II + apocynin) group; F. Agonist (TBCA) group; G. Agonist + Treatment (TBCA + picroside II) group; H. Vehicle (DMSO) group. *P<0.05 compared to group B. The data were compared with one-way ANOVA; Values are presented as means ± SD; n = 5. (ZIP) [file pone.0174414.s003.zip › S3 Fig/B2.tif]

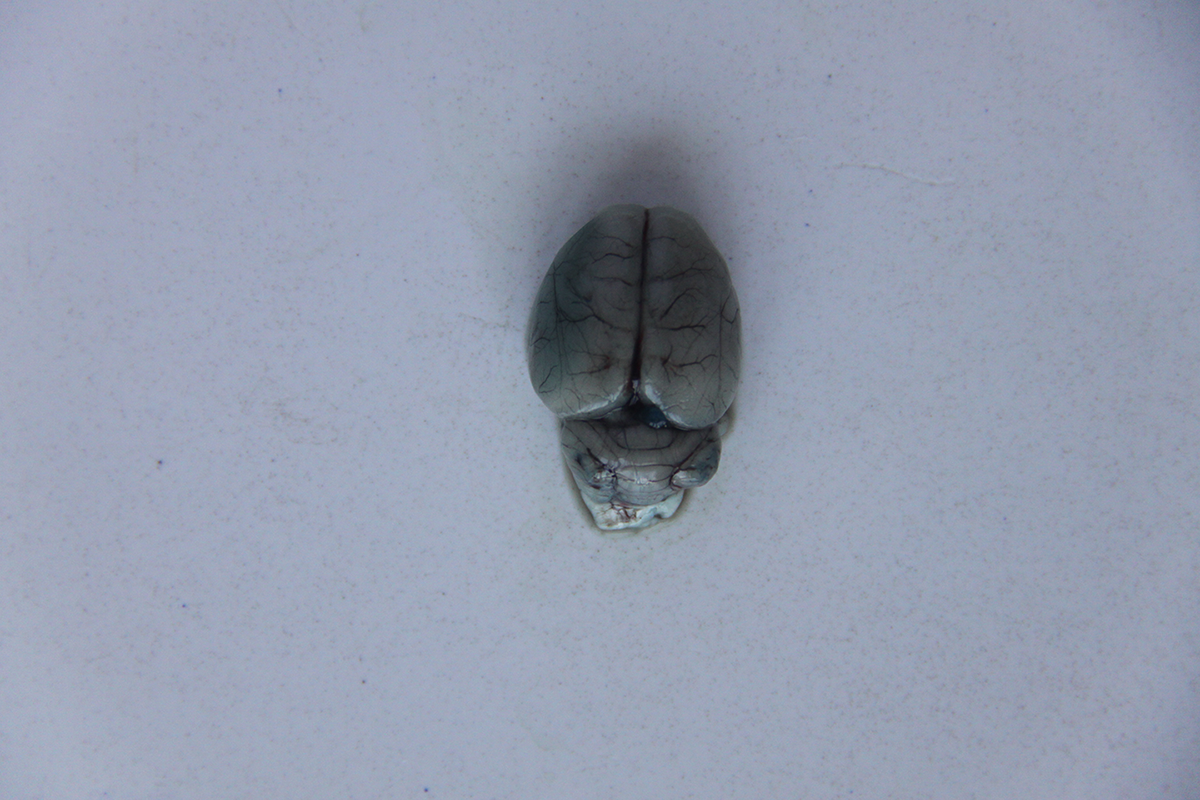

Supplement: S3 Fig — A. Sham group; B. Model group; C. Treatment (picroside II) group; D. Positive control (apocynin) group; E. Treatment + Positive control (picroside II + apocynin) group; F. Agonist (TBCA) group; G. Agonist + Treatment (TBCA + picroside II) group; H. Vehicle (DMSO) group. *P<0.05 compared to group B. The data were compared with one-way ANOVA; Values are presented as means ± SD; n = 5. (ZIP) [file pone.0174414.s003.zip › S3 Fig/C1.tif]

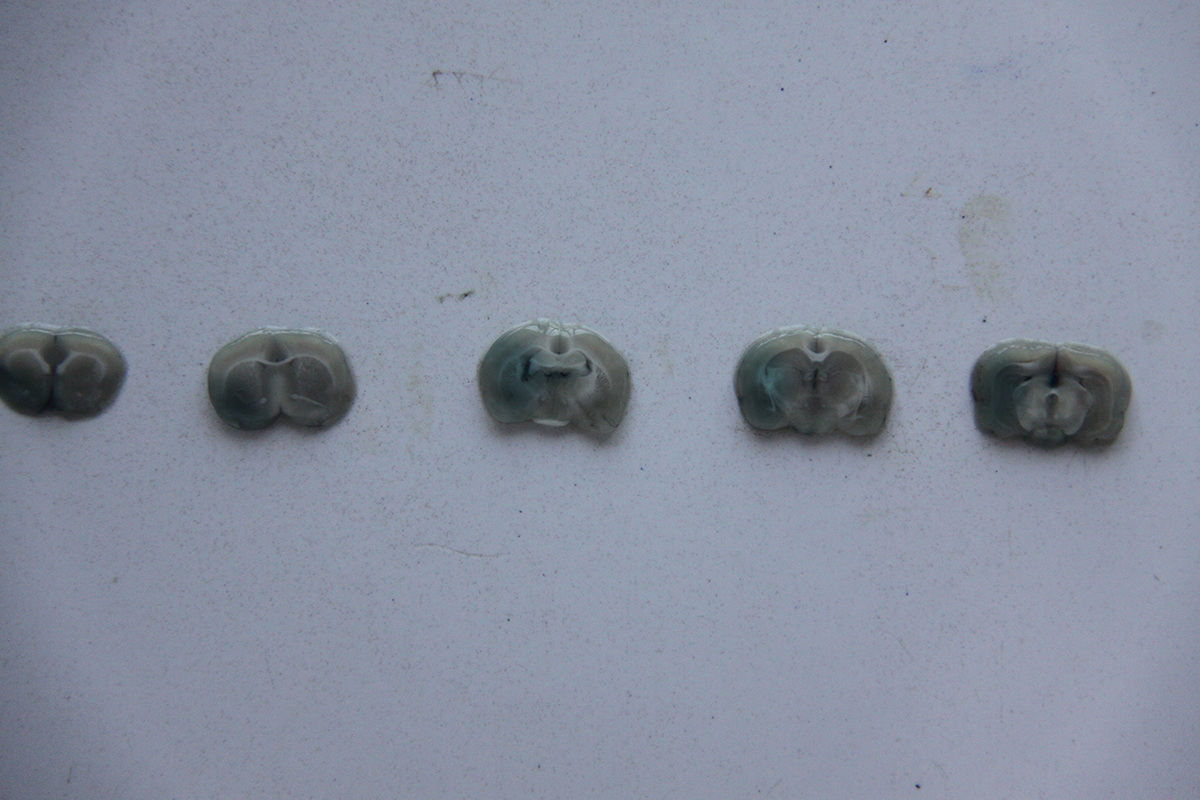

Supplement: S3 Fig — A. Sham group; B. Model group; C. Treatment (picroside II) group; D. Positive control (apocynin) group; E. Treatment + Positive control (picroside II + apocynin) group; F. Agonist (TBCA) group; G. Agonist + Treatment (TBCA + picroside II) group; H. Vehicle (DMSO) group. *P<0.05 compared to group B. The data were compared with one-way ANOVA; Values are presented as means ± SD; n = 5. (ZIP) [file pone.0174414.s003.zip › S3 Fig/C2.tif]

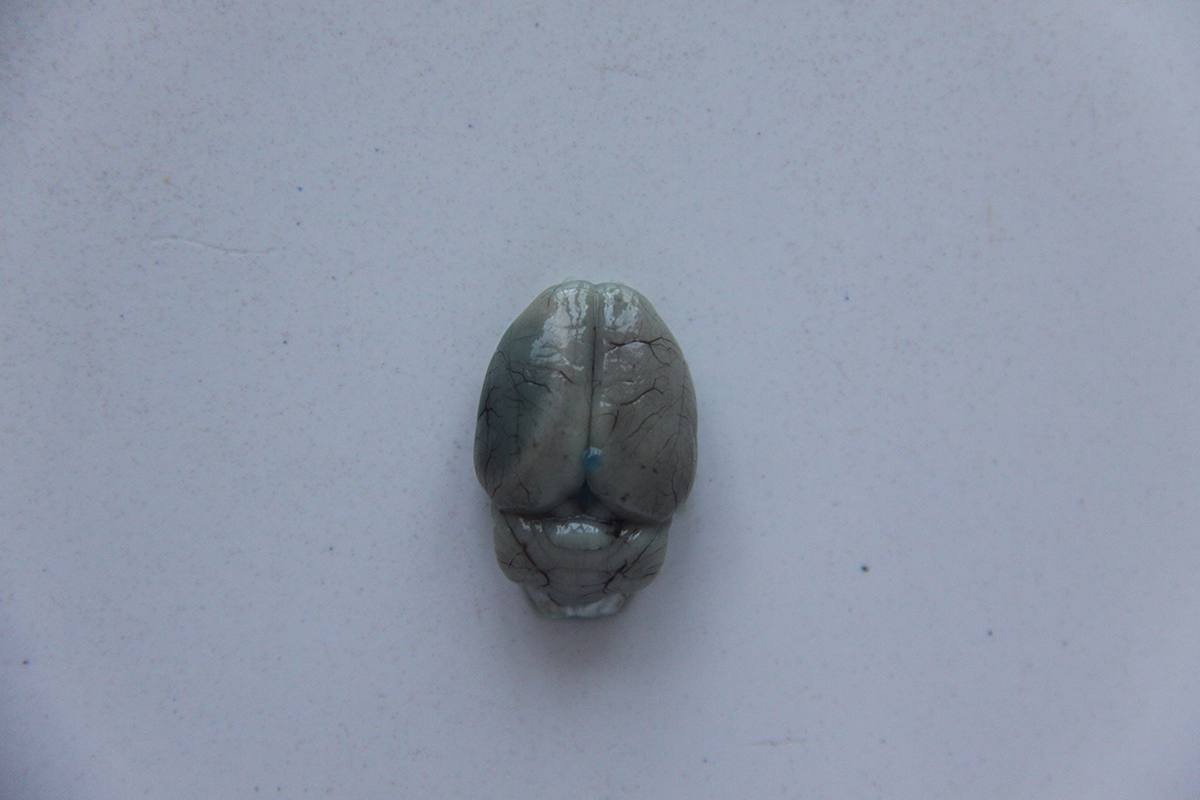

Supplement: S3 Fig — A. Sham group; B. Model group; C. Treatment (picroside II) group; D. Positive control (apocynin) group; E. Treatment + Positive control (picroside II + apocynin) group; F. Agonist (TBCA) group; G. Agonist + Treatment (TBCA + picroside II) group; H. Vehicle (DMSO) group. *P<0.05 compared to group B. The data were compared with one-way ANOVA; Values are presented as means ± SD; n = 5. (ZIP) [file pone.0174414.s003.zip › S3 Fig/D1.tif]

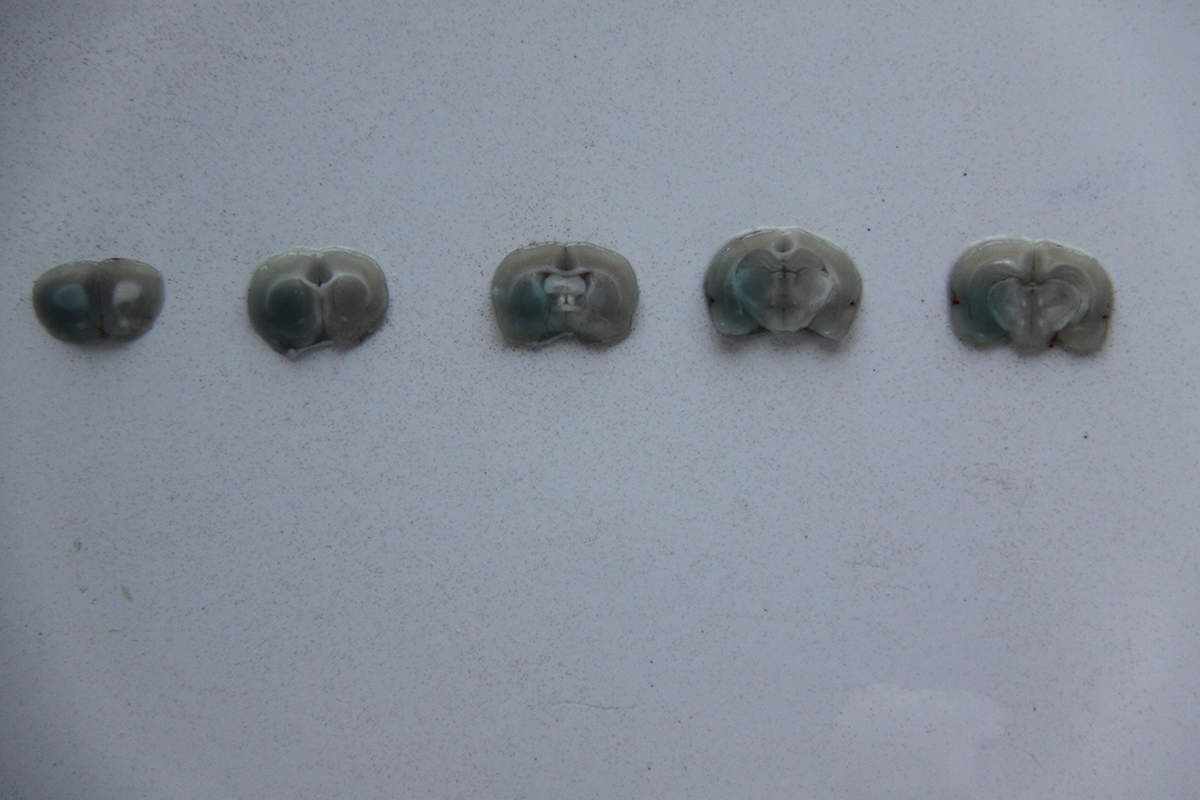

Supplement: S3 Fig — A. Sham group; B. Model group; C. Treatment (picroside II) group; D. Positive control (apocynin) group; E. Treatment + Positive control (picroside II + apocynin) group; F. Agonist (TBCA) group; G. Agonist + Treatment (TBCA + picroside II) group; H. Vehicle (DMSO) group. *P<0.05 compared to group B. The data were compared with one-way ANOVA; Values are presented as means ± SD; n = 5. (ZIP) [file pone.0174414.s003.zip › S3 Fig/D2.tif]

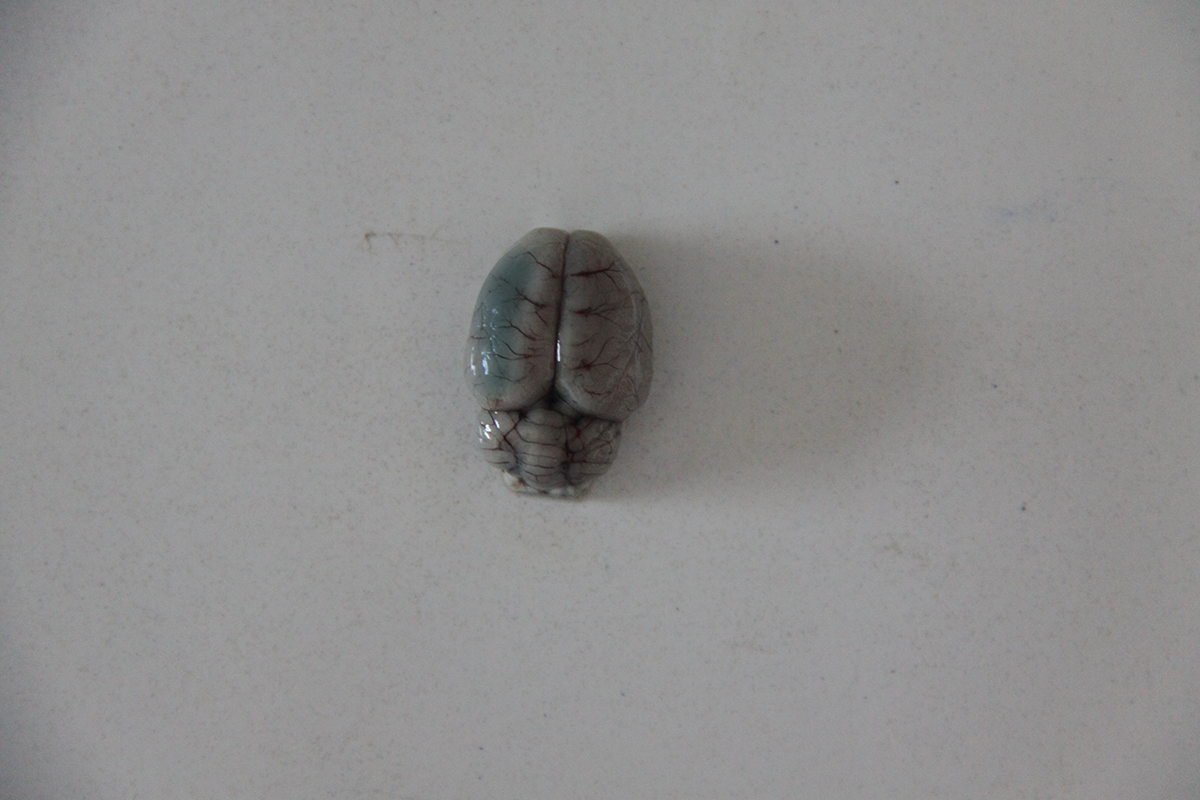

Supplement: S3 Fig — A. Sham group; B. Model group; C. Treatment (picroside II) group; D. Positive control (apocynin) group; E. Treatment + Positive control (picroside II + apocynin) group; F. Agonist (TBCA) group; G. Agonist + Treatment (TBCA + picroside II) group; H. Vehicle (DMSO) group. *P<0.05 compared to group B. The data were compared with one-way ANOVA; Values are presented as means ± SD; n = 5. (ZIP) [file pone.0174414.s003.zip › S3 Fig/E1.tif]

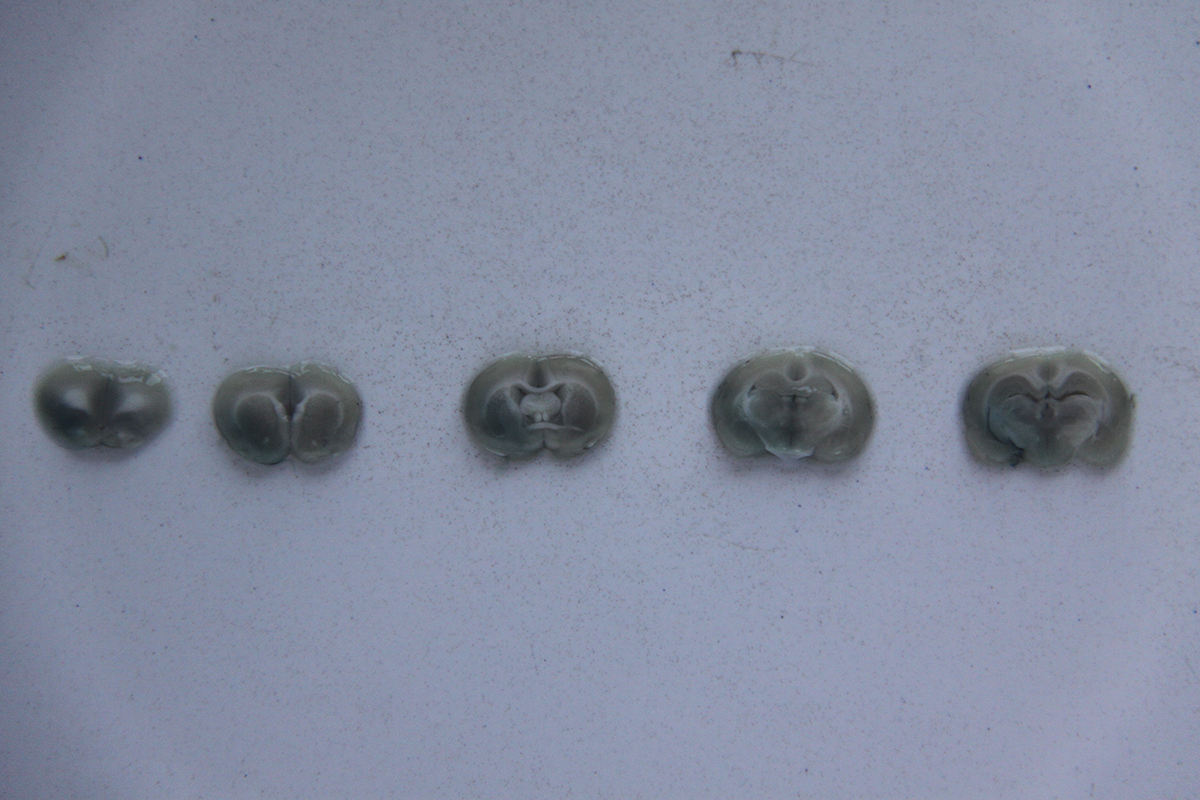

Supplement: S3 Fig — A. Sham group; B. Model group; C. Treatment (picroside II) group; D. Positive control (apocynin) group; E. Treatment + Positive control (picroside II + apocynin) group; F. Agonist (TBCA) group; G. Agonist + Treatment (TBCA + picroside II) group; H. Vehicle (DMSO) group. *P<0.05 compared to group B. The data were compared with one-way ANOVA; Values are presented as means ± SD; n = 5. (ZIP) [file pone.0174414.s003.zip › S3 Fig/E2.tif]

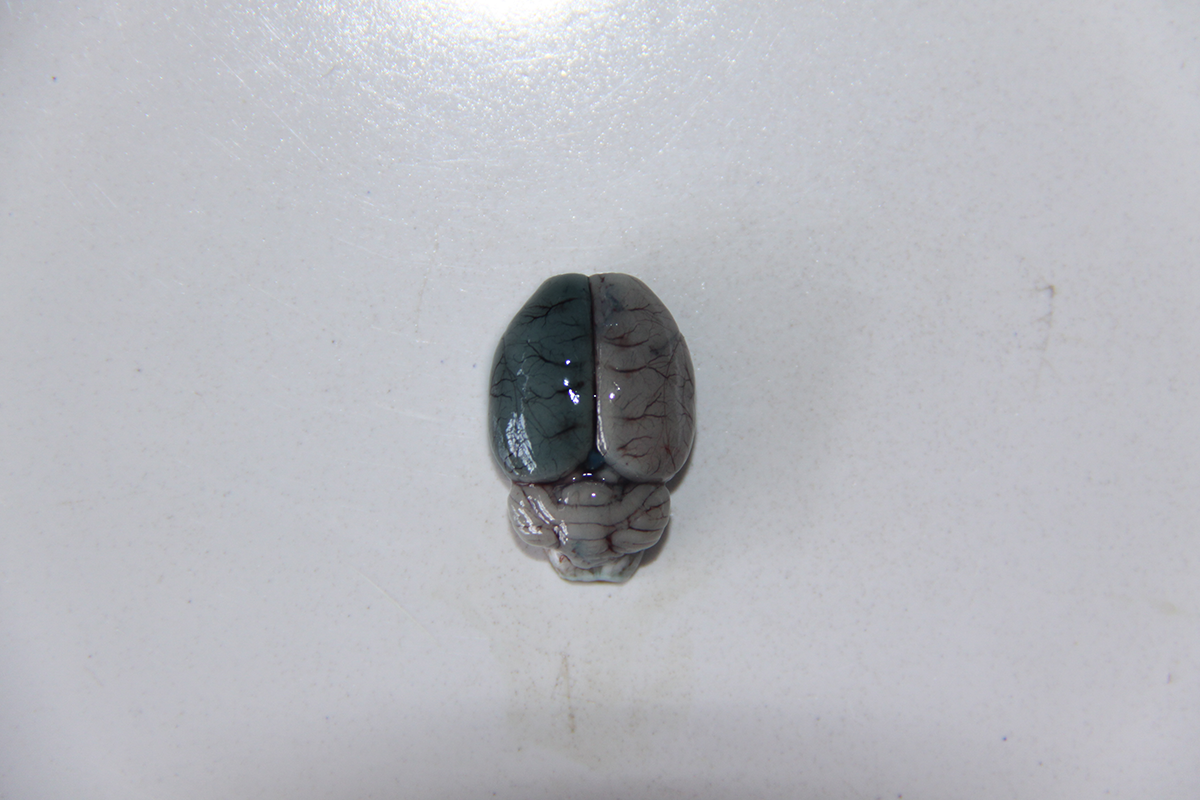

Supplement: S3 Fig — A. Sham group; B. Model group; C. Treatment (picroside II) group; D. Positive control (apocynin) group; E. Treatment + Positive control (picroside II + apocynin) group; F. Agonist (TBCA) group; G. Agonist + Treatment (TBCA + picroside II) group; H. Vehicle (DMSO) group. *P<0.05 compared to group B. The data were compared with one-way ANOVA; Values are presented as means ± SD; n = 5. (ZIP) [file pone.0174414.s003.zip › S3 Fig/F1.tif]

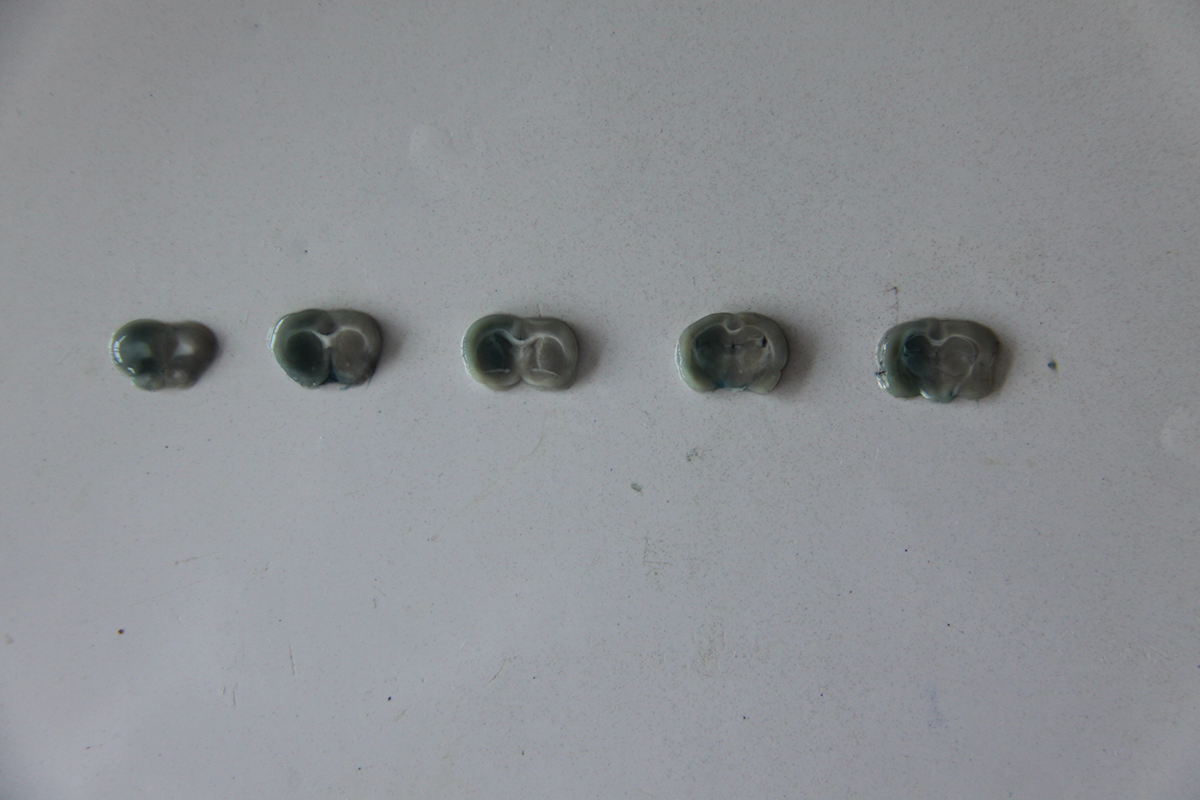

Supplement: S3 Fig — A. Sham group; B. Model group; C. Treatment (picroside II) group; D. Positive control (apocynin) group; E. Treatment + Positive control (picroside II + apocynin) group; F. Agonist (TBCA) group; G. Agonist + Treatment (TBCA + picroside II) group; H. Vehicle (DMSO) group. *P<0.05 compared to group B. The data were compared with one-way ANOVA; Values are presented as means ± SD; n = 5. (ZIP) [file pone.0174414.s003.zip › S3 Fig/F2.tif]

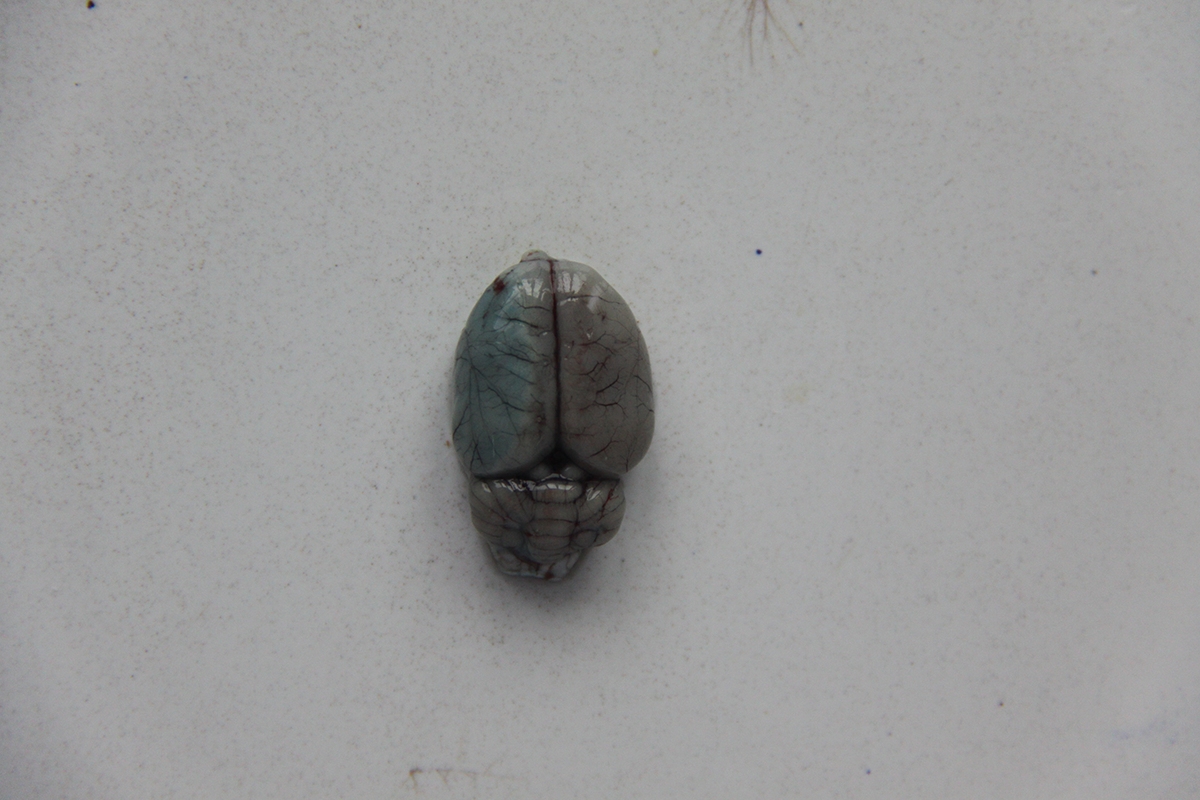

Supplement: S3 Fig — A. Sham group; B. Model group; C. Treatment (picroside II) group; D. Positive control (apocynin) group; E. Treatment + Positive control (picroside II + apocynin) group; F. Agonist (TBCA) group; G. Agonist + Treatment (TBCA + picroside II) group; H. Vehicle (DMSO) group. *P<0.05 compared to group B. The data were compared with one-way ANOVA; Values are presented as means ± SD; n = 5. (ZIP) [file pone.0174414.s003.zip › S3 Fig/G1.tif]

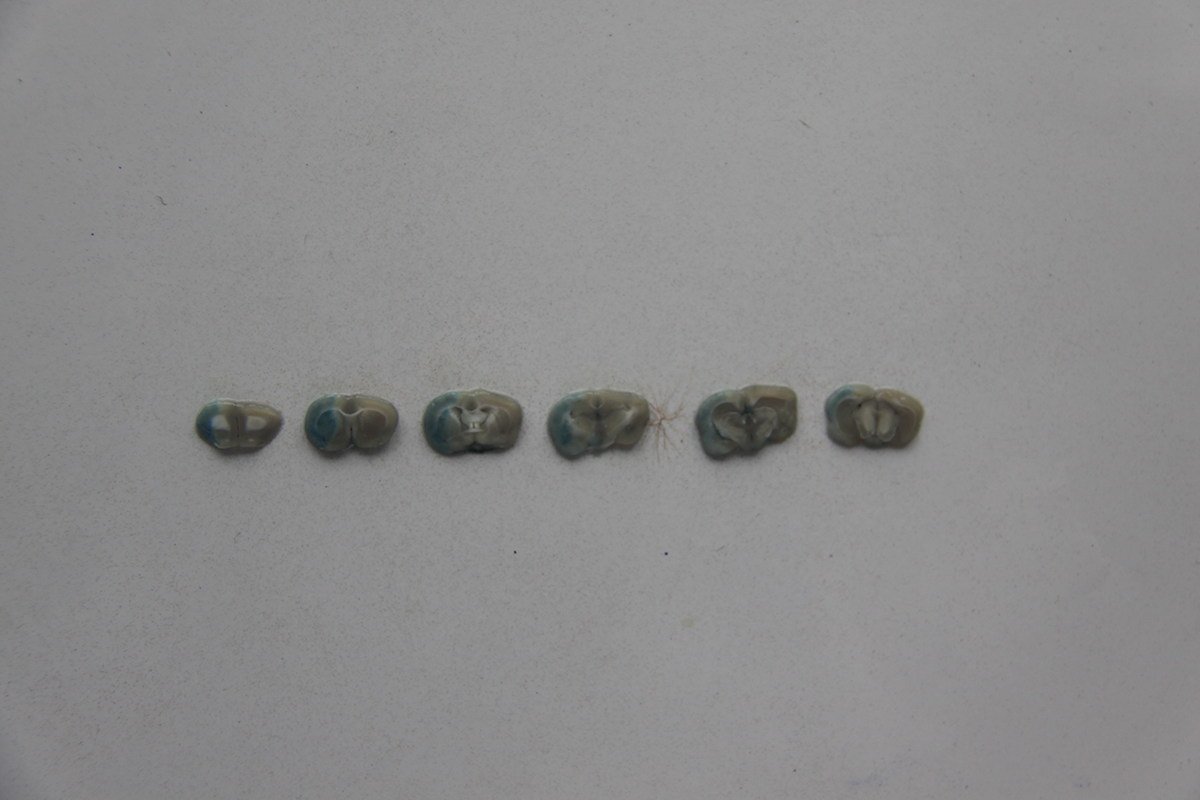

Supplement: S3 Fig — A. Sham group; B. Model group; C. Treatment (picroside II) group; D. Positive control (apocynin) group; E. Treatment + Positive control (picroside II + apocynin) group; F. Agonist (TBCA) group; G. Agonist + Treatment (TBCA + picroside II) group; H. Vehicle (DMSO) group. *P<0.05 compared to group B. The data were compared with one-way ANOVA; Values are presented as means ± SD; n = 5. (ZIP) [file pone.0174414.s003.zip › S3 Fig/G2.tif]

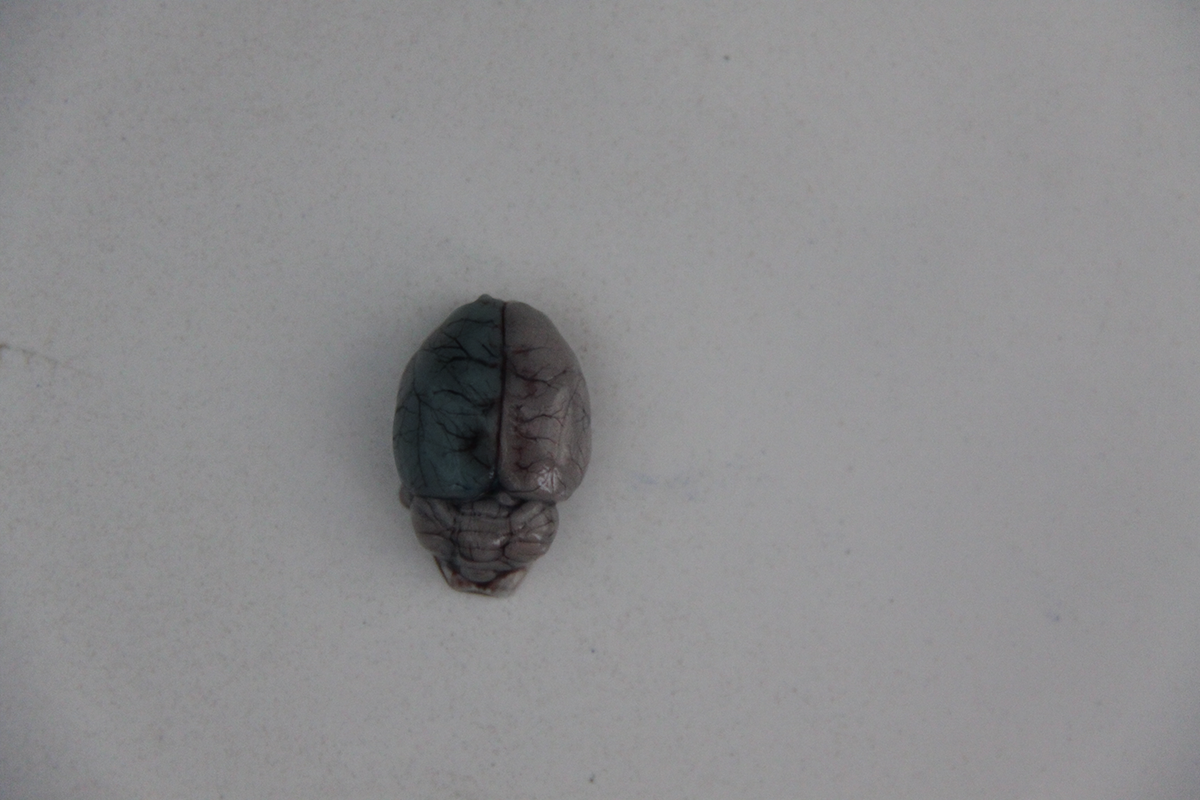

Supplement: S3 Fig — A. Sham group; B. Model group; C. Treatment (picroside II) group; D. Positive control (apocynin) group; E. Treatment + Positive control (picroside II + apocynin) group; F. Agonist (TBCA) group; G. Agonist + Treatment (TBCA + picroside II) group; H. Vehicle (DMSO) group. *P<0.05 compared to group B. The data were compared with one-way ANOVA; Values are presented as means ± SD; n = 5. (ZIP) [file pone.0174414.s003.zip › S3 Fig/H1.tif]

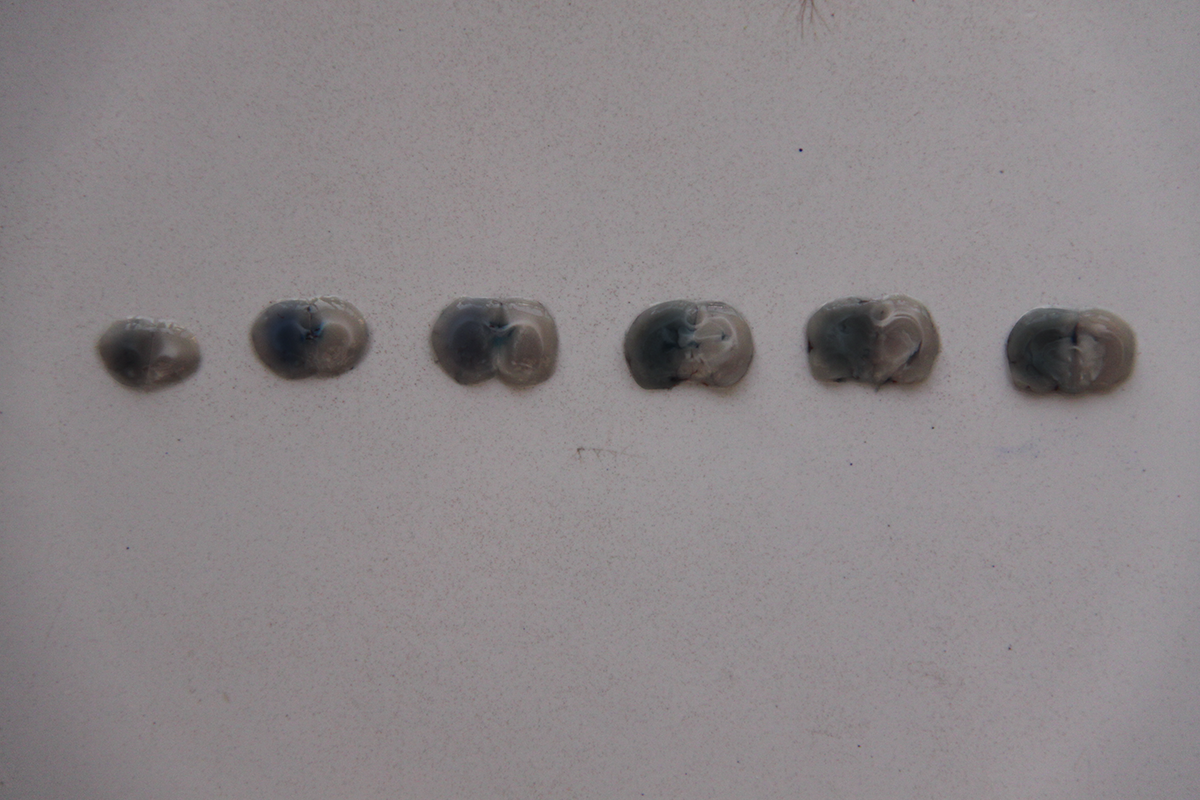

Supplement: S3 Fig — A. Sham group; B. Model group; C. Treatment (picroside II) group; D. Positive control (apocynin) group; E. Treatment + Positive control (picroside II + apocynin) group; F. Agonist (TBCA) group; G. Agonist + Treatment (TBCA + picroside II) group; H. Vehicle (DMSO) group. *P<0.05 compared to group B. The data were compared with one-way ANOVA; Values are presented as means ± SD; n = 5. (ZIP) [file pone.0174414.s003.zip › S3 Fig/H2.tif]
